# Highly Active and Stable Ni/La-Doped Ceria Material for Catalytic CO_2_ Reduction by Reverse Water-Gas Shift Reaction

**DOI:** 10.1021/acsami.2c11248

**Published:** 2022-11-02

**Authors:** Consuelo Alvarez-Galvan, Pablo G. Lustemberg, Freddy E. Oropeza, Belén Bachiller-Baeza, Martin Dapena Ospina, María Herranz, Jesús Cebollada, Laura Collado, José M. Campos-Martin, Víctor A. de la Peña-O’Shea, José A. Alonso, M. Verónica Ganduglia-Pirovano

**Affiliations:** † Instituto de Catálisis y Petroleoquímica (CSIC), Cantoblanco, Madrid28049, Spain; ‡ Instituto de Física Rosario (IFIR), CONICET-UNR, Rosario, Santa Fe2000EZP, Argentina; § Photoactivated Processes Unit, IMDEA Energy Institute, Avda. Ramón de la Sagra 3, Móstoles, Madrid28935, Spain; ∥ Instituto de Ciencia de Materiales de Madrid (CSIC), Cantoblanco, Madrid28049, Spain

**Keywords:** rWGS, nickel, CeO_2_, La-doping, DFT calculations

## Abstract

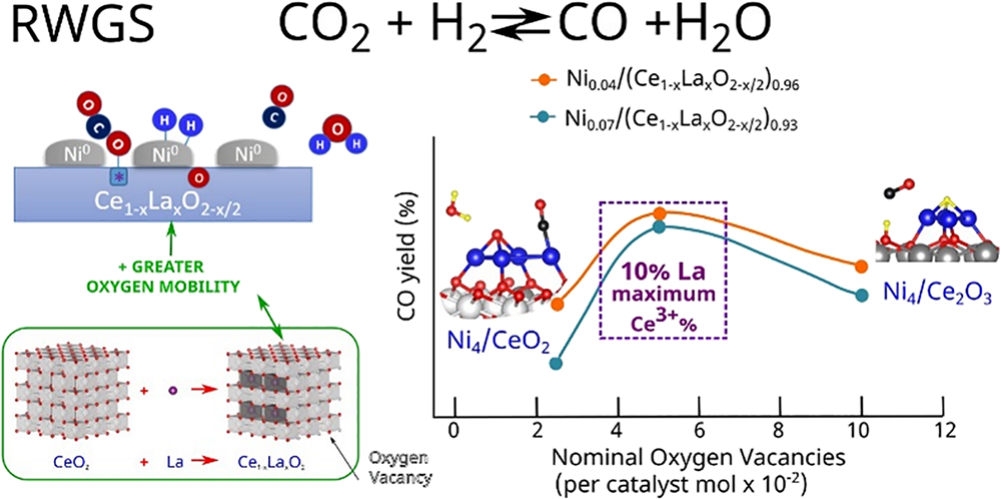

The design of an active, effective, and economically viable catalyst for CO_2_ conversion into value-added products is crucial in the fight against global warming and energy demand. We have developed very efficient catalysts for reverse water-gas shift (rWGS) reaction. Specific conditions of the synthesis by combustion allow the obtention of macroporous materials based on nanosized Ni particles supported on a mixed oxide of high purity and crystallinity. Here, we show that Ni/La-doped CeO_2_ catalysts—with the “right” Ni and La proportions—have an unprecedented catalytic performance per unit mass of catalyst for the rWGS reaction as the first step toward CO_2_ valorization. Correlations between physicochemical properties and catalytic activity, obtained using a combination of different techniques such as X-ray and neutron powder diffraction, Raman spectroscopy, in situ near ambient pressure X-ray photoelectron spectroscopy, electron microscopy, and catalytic testing, point out to optimum values for the Ni loading and the La proportion. Density functional theory calculations of elementary steps of the reaction on model Ni/ceria catalysts aid toward the microscopic understanding of the nature of the active sites. This finding offers a fundamental basis for developing economical catalysts that can be effectively used for CO_2_ reduction with hydrogen. A catalyst based on Ni_0.07_/(Ce_0.9_La_0.1_O_*x*_)_0.93_ shows a CO production of 58 × 10^–5^ mol_CO_·g_cat_^–1^·s^–1^ (700 °C, H_2_/CO_2_ = 2; selectivity to CO > 99.5), being stable for 100 h under continuous reaction.

## 1 Introduction

Carbon dioxide is the most important greenhouse gas, and it is necessary to reduce its emissions by the progressive replacement of fossils by renewable sources. At present, numerous efforts have been made with the aim to consider this waste as a resource, investing in the development of new technologies that drive its recycling.^1^ Only reactions that produce fuels or bulk chemicals can be considered as real solutions to substantially reduce CO_2_ emissions using large-volume sources such as those from thermoelectric power stations or from biomass-waste gasification.^2,3^ In this scenario, reverse water-gas shift (rWGS) reaction, in which CO_2_ is catalytically reduced with (preferable) renewable H_2_ to CO, would constitute the first stage in CO_2_ valorization by Fischer–Tropsch and/or methanol syntheses. This is a slightly endothermic reaction, with the equilibrium shifted toward the products at high reaction temperatures (∼700–800 °C). Furthermore, elevated temperatures should decrease the formation of undesirable byproducts such as CH_4_, produced by the competing exothermic methanation reaction, and carbon, formed by the Boudouard reaction. The use of a catalyst that allows high conversion, high selectivity, and durability under harsh operation conditions is very necessary, but its design is challenging.^4,5^ As reported in the literature, the catalytic activity for rWGS is enhanced by using both an active phase in the metallic state,^6^ such as transition metals like nickel, which is known to facilitate spillover of dissociated hydrogen from the metal and has a lower price compared with noble metals,^7^ and a reducible support with oxygen vacancies, which promote CO_2_ adsorption and activation.^8−11^ Among different reducible supports, CeO_2_ appears to be one of the most efficient in activating CO_2_ molecules at oxygen vacancies formed under high reduction temperatures,^12^ evidencing that the metal–support interface is crucial for catalytic activity.^13,14^ Partially reduced ceria as a support of transition metal nanoparticles stabilizes their dispersion^15^ and improves the reducibility of the system due to its excellent oxygen mobility.^16^

Operando spectroscopic techniques, such as DRIFTS, have been used to unravel the reaction mechanism, oxidation state of active phases, reaction intermediates, and spectators.^17^ Note that reaction mechanism studies by DRIFTS have been performed at medium reaction temperatures (∼300–400 °C), for which the methanation reaction is thermodynamically more favorable. At higher reaction temperatures, intermediates and spectators are desorbed; thus, their detection is not possible. The most accepted rWGS reaction mechanism over oxide-supported metal catalysts suggests that the dissociative adsorption of H_2_ takes place on the metal particles and that the adsorption of CO_2_ occurs on the reducible oxide support, which is followed by its reduction to CO(g). At high temperatures and using Ni as a metal phase, among other metals, the rate-limiting step for the rWGS reaction is considered to be water formation, produced by hydrogen migration from the supported metal particles to lattice oxygen atoms of the support.^17−19^

Although numerous works have addressed the catalytic thermochemical CO_2_ reduction with H_2_, a fundamental understanding of how to control selectivity is still poor, and the design of active and efficient rWGS catalysts remains a challenging task.^20^

In this work, we demonstrate the result of fine-tuning the metal loading and dopant proportion in Ni_*y*_(Ce_1–*x*_La_*x*_O_2–*x*/2_)_1–*y*_ catalysts to increase the rWGS activity and control the selectivity. A certain proportion of rare earths as dopants of ceria produce supports with different amounts of oxygen vacancies, which would have an influence on CO_2_ adsorption and oxygen mobility during the reaction. Among the possible methods to prepare these ceria-supported Ni catalysts, we have selected the solution combustion synthesis (SCS) method. Using a patented specific protocol that includes, among other conditions, a controlled ratio between fuel and metal precursors, a porous and finely particulate metal oxides materials with very high macroporosity and the formation of Ni particles in the metallic state are obtained.^21−23^ These critical points help to promote heat and mass transport and prevent the formation of hot-spots during the catalytic reaction. A deep characterization using X-ray and neutron diffraction, Raman spectroscopy, in situ NAP-XPS, and electron microscopy, in combination with DFT calculations, was performed and correlated with the reaction performance, highlighting the key parameters that control reactivity. These findings can be useful in the successful creation of catalysts involving H–H and C=O bond dissociation, opening the possibility for exciting chemistry.

## 2 Experimental Section

### 2.1 Catalyst Preparation

Samples based on Ni_*y*_/(Ce_1–*x*_La_*x*_O_2–*x*/2_)_1–*y*_ (*x* = 0, 0.05, 0.1, and 0.2 and *y* = 0.01, 0.04, 0.07, and 0.1, see Table S1) were prepared by the SCS method. Hereinafter, specific samples will be denoted by the nickel loading and the La-dopant proportion, e.g. Ni0.07/Ce0.9La0.1 refers to a sample with a nickel loading of 7% and 10% La content, i.e., Ni_0.07_/(Ce_0.9_La_0.1_O_1.95_)_0.93_. In a typical synthesis, nitrates of Ni, Ce, and La were dissolved in deionized water, and then some amount of fuel was added.^21^ The solution was heated on a hot plate at 310 °C. Once most of the water was removed, a viscous gel was formed. Then, auto-ignition with flame was produced, leading, in a single step, to a fine particulate powder consisting of Ni nanoparticles supported on the corresponding doped ceria mixed oxide.^23^

### 2.2 Catalyst Characterization

The identification of the crystalline phases was performed by laboratory X-ray diffraction (XRD) patterns, collected on a Bruker D5 diffractometer with KαCu (λ = 1.5418 Å) radiation. The Rietveld^24^ profile refinement procedure was used to treat XRD data, with FULLPROF software.^25^ The line shape of the peaks was generated with a pseudo-Voigt function. In the final run, the following profile parameters were refined: scale factor, background coefficients, zero-point error, and pseudo-Voigt corrected for asymmetry parameters. The structural parameters refined were the isotropic thermal factors for all the atoms and occupancy factor for O. Oxygen positions in oxide networks were determined by neutron powder diffraction (NPD). The patterns were collected at the high-resolution D2B neutron diffractometer of ILL (Grenoble-France), with the high-flux mode and a counting time of 2 h. The sample was introduced in a vanadium holder. A wavelength of 1.594 Å was selected from a Ge monochromator; the temperature during the analysis was 295 K. The NPD patterns were also refined by the Rietveld method,^24^ as described before. The coherent scattering lengths for Ce, La, Ni, and O atoms were 4.84, 8.24, 10.30, and 5.803 fm, respectively.

Raman spectra were obtained using a Raman microscope spectrometer (Renishaw) with a laser beam emitting at 532 nm and 100 mW. The photons scattered by the sample were dispersed by a 1800 lines/mm grating monochromator and, at the same time, collected on a CCD camera. The objective was set at 50×.

For TEM studies, the samples were suspended in *n*-butyl alcohol or ethanol and ultrasonically dispersed. A few drops of the resulting suspension were deposited on a carbon-coated grid. The analyses were carried out with a JEOL JEM 3000F microscope working at 300 kV (double tilt: ±20°) (point resolution: 0.17 nm), fitted with an X-ray energy-dispersive spectroscopy (XEDS) microanalysis system (OXFORD INCA), and an ENFINA spectrometer with an energy resolution of 1.3 eV.

High-resolution field emission scanning electron microscopy (FE-SEM) images were collected in a FEI Nova NanoSEM 230.

The BET surface area of the samples was determined from N_2_ adsorption–desorption isotherms at −196 °C. These analyses were performed with a Micromeritics ASAP 2000 apparatus on samples previously outgassed at 140 °C overnight.

Mercury intrusion porosimetry experiments were performed using AutoPore IV 9510 equipment. Raising the pressure from vacuum to 200 MPa, pore diameters from 200 μm to 7.5 nm can be determined. This technique was also used to evaluate the open porosity of the samples.

Temperature-programmed reduction (TPR) experiments were carried out in a Micromeritics TPD/TPR 2900. The samples were pretreated under helium at 110 °C for 15 min. The reduction profile was recorded by heating the sample from room temperature to 800 °C at a rate of 10 °C·min^–1^ under a H_2_/Ar (10% v/v) flow.

To determine Ni dispersion, pulse chemisorption was performed by an AutoChem 2920 station (Micromeritics). The samples were placed in a U-shaped quartz reactor with an inner diameter of 5 mm and pretreated under 10% H_2_ in Ar at 600 °C for 30 min. Then, the samples were treated with Ar at 620 °C under an argon flow for 30 min before further cooling for chemisorption in order to clean the surface and to avoid the presence of residual adsorbed hydrogen. Pulse chemisorption experiments of pure H_2_ were performed at 0 °C using a cryocooler. Ar was used as the carrier gas until stable peaks were observed.

In situ studies by near ambient pressure X-ray photoelectron spectroscopy (NAP-XPS) were carried out employing a lab-based spectrometer (SPECS GmbH, Berlin) using a monochromated AlKα1 source (*h*ν = 1486.6 eV) operating at 50 W. The X-rays are microfocused to give 300 μm a spot size on the samples. The spectrometer analyzer is a SPECS PHOIBOS 150 NAP, a true 180° hemispherical energy analyzer with 150 mm mean radius. The entrance to the analyzer is a nozzle with a 300 μm-diameter orifice. The total energy resolution of the measurements was about 0.50 eV. The binding energy (BE) was calibrated against the Au Fermi level. Samples were exposed to a 2 mbar total pressure of a 2:1 H_2_:CO_2_ (molar ratio) reactive mixture, and the temperature was increased from room temperature to 600 °C. Each temperature was dwelled for 15 min before taking the spectra.

Temperature-programmed desorption (TPD) measurements were performed using an AUTOCHEM II 2910 instrument (Micromeritics), equipped with a flow-through quartz reactor and a thermal conductivity detector (TCD). For CO_2_-TPD tests, the catalyst (25 mg) was first pretreated as follows: degasification under He at 500 °C for 1 h at a ramp of 10 °C min^–1^; cooling down to 40 °C in a He flow; saturation with CO_2_ (5% CO_2_ in He) at 80 °C for 30 min; and flushing with He at 80 °C for 30 min to remove the weakly physisorbed CO_2_. Then, the CO_2_-TPD test was carried out by heating the catalyst from room temperature to 800 °C with a ramp of 10 °C·min^–1^ under a He flow.

### 2.3 Theoretical Calculations

Spin-polarized DFT calculations were performed to gain insights into the roles of the metal and oxide phases of low-loaded nickel-ceria model catalysts in the activation of CO_2_ as well as the H_2_ dissociation and subsequent H diffusion that ultimately leads to the formation of CO and H_2_O. Previous experimental results^21,22^ indicate that the system consists of nickel metal nanoparticles supported on a La-doped ceria with different degrees of reduction. We chose a flat Ni_4_ cluster structure supported on the fully oxidized CeO_2_(111) and on the fully reduced Ce_2_O_3_(0001) surfaces as representative models of low-loaded ceria-supported nickel catalysts, which illustrate accurately many of the essential atomic-scale features that control the catalyst stability, electronic structure, and surface reactivity, as described below. The stability of the flat Ni_4_ cluster on the CeO_2_(111) and that of a pyramidal one are comparable.^26,27^ The results are compared with those for the extended Ni(111) surface.

The VASP code (vasp site, http://www.vasp.at; version vasp.5.3.5)^28,29^ was used to perform all electronic structure calculations. The projector augmented wave (PAW) method^30^ with a plane-wave cutoff energy of 415 eV was used for the explicit description of the valence electrons of Ce (4f, 5s, 5p, 5d, 6s), O (2s, 2p), and Ni (3p, 3d, 4s), while the rest of the electrons are included in the description of the atomic nuclei. In this work, the DFT + U framework proposed by Dudarev et al.^31^ (*U*_eff_ = *U* – *J* = 4.5 eV for the Ce 4f electrons) was used together with the generalized gradient approximation (GGA) proposed by Perdew, Burke, and Ernzerhof (PBE).^32^ The energies and forces were calculated with an accuracy of 10^–6^ eV and 10^–2^ eV/Å, respectively.

The Ni_4_/CeO_2_(111), Ni_4_/Ce_2_O_3_(0001), and Ni(111) model catalysts were modeled using 3 × 3 surface unit cells. The ceria bulk equilibrium lattice constants used to create the supercells were for CeO_2_ (5.485 Å) and for Ce_2_O_3_ (*a*_0_/*c*_0_ = 3.917/6.182 Å) with internal parameters *u*_Ce_/*u*_O_ = 0.2471/0.6448. In the case of the Ni_4_/CeO_2_(111) and Ni_4_/Ce_2_O_3_(0001) surfaces, slabs of two CeO_2_ (O-Ce-O) tri-layers and two Ce_2_O_3_ (O-Ce-O-Ce-O) quintuple-layers, respectively, were considered. For the extended clean Ni(111) surface, a five layer-thick slab with the PBE optimized lattice constant (fcc Ni: 3.52 Å) was used. In all surface models, consecutive slabs were separated by at least a 12 Å-thick vacuum layer to avoid interaction between the slabs and their periodic images.

The Brillouin zone was sampled with a Monkhorst-Pack mesh of 2 × 2 × 1 and 5 × 5 × 1 *k*-points for ceria-based systems and Ni(111) surfaces, respectively. During the optimization process, all atoms were relaxed except those at the bottom CeO_2_ (Ce_2_O_3_) layers and the last two Ni(111) layers, whose positions correspond to those of the corresponding bulk-truncated slabs.

We define the adsorption energy as *E*_ads_ = *E*[Ads/Cat] – *E*[Cat.] – *E*[Ads_gas_], where *E*[Ads/Cat] is the total energy of the molecular or dissociative state of the adsorbate species (CO_2_ and H_2_) on the catalyst, *E*[Cat.] is the total energy of the surface without the adsorbate, and *E*[Ads_gas_] is the energy of the adsorbate molecule in the gas phase.

We employed the climbing image nudged elastic band method (CI-NEB),^33^ as we have reported in a previous work.^27^ It was verified that all TS have a single imaginary frequency and that the images adjacent to the TS relax either toward the initial state or the final state of the reaction path.

### 2.4 Catalytic Reactions

A continuous flow fixed-bed quartz tubular reactor (4 mm, inner diameter) was used to perform the rWGS reaction under atmospheric pressure. The catalyst (with a particle size between 50 and 100 μm) was activated under the reactive feed up to 700 °C. The space velocity was 3 × 10^5^ mL_N_·h^–1^·g^–1^ or 6 × 10^5^ mL_N_·h^–1^·g^–1^, and the vol. feed composition was 60% H_2_, 30% CO_2_, and 10% N_2_. The outlet gas stream was analyzed online by gas chromatography (HP 6890), equipped with a column Carboxen 1010 PLOT (SUPELCO) and with a thermal conductivity detector. N_2_ was used as an inert standard for quantification. The catalysts were tested for about 6 h between 500 and 700 °C. A durability study was also performed with the most active and selective catalyst composition at 700 °C (for 96 h of reaction in continuous operation). The kinetic study was performed with the catalyst Ni0.07/Ce0.9La0.1 at a space velocity of 428,571 mL_N_·h^–1^·g^–1^ (H_2_/CO_2_ = 2 molar and 10% N_2_) at 700, 725, 750, and 775 °C for the calculation of the activation energy. The determination of reaction orders for CO_2_ and H_2_ was carried out at 750 ° C and under the following H_2_/CO_2_ ratios: 1.5, 2, 2.5, and 3.

## 3 Results and Discussion

**Figure 1:**
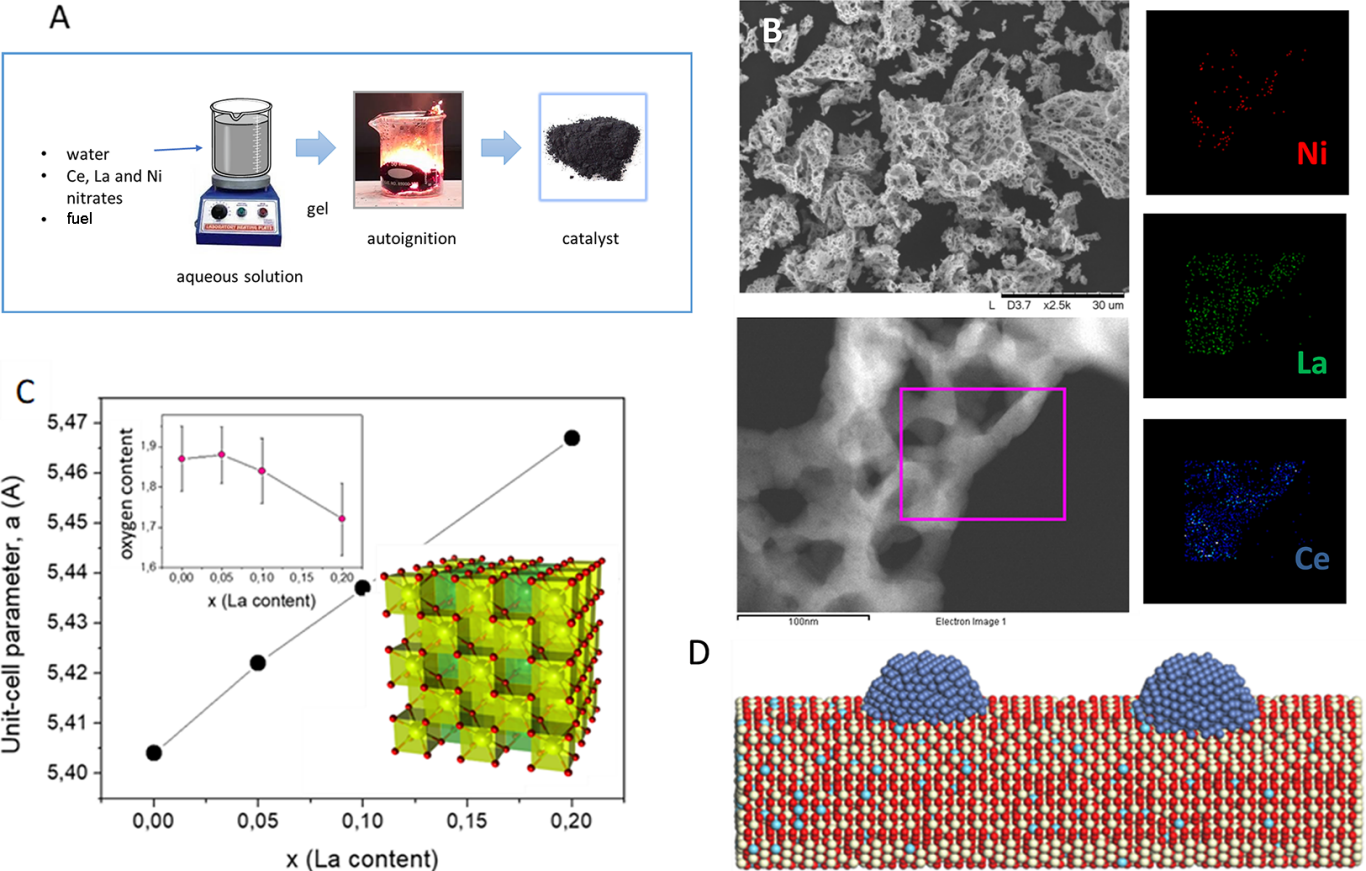
(A) Scheme of the solution combustion method synthesis procedure. (B) SEM (up) and STEM images of the Ni0.07/Ce0.9La0.1 catalyst and mapping analysis for Ni, La, and Ce elements. (C) Unit-cell parameters as a function of La doping level in Ni_0.07_/(Ce_1–*x*_La_*x*_O_2–*x*/2_)_0.93_ by X-ray diffraction and Rietveld refinement. The inset shows a sketch of the fluorite structure, enhancing the cubic coordination of CeO_8_ polyhedra and the partial occupancy of both Ce (light green), La (green), and O (red) atoms. The upper inset shows the oxygen content as a function of La proportion. (D) Illustration of nickel-supported La-doped ceria structures Ce (beige), O (red), La (light blue), and Ni (blue).

To determine the effect of La doping in the rWGS catalytic performance of Ni/CeO_2_ systems, a series of Ni_*y*_/(Ce_1–*x*_La_*x*_O_2–*x*/2_)_1–*y*_ (*x* = 0, 0.05, 0.1, and 0.2; *y* = 0.04 and 0.07) were prepared using a patented solution combustion method. X-ray powder diffraction (XRD) measurements (Figure S1) show in all cases that the presence of La does not introduce changes in the fluorite structure of bare CeO_2._ The Rietveld refinement for the complete La doping series (*x* = 0, 0.05, 0.1, and 0.2) confirms that the unit-cell parameter regularly increases with the La doping level (*x*), assessing the effect of the insertion of the lanthanide cation into the ceria lattice (Figure 1C and Figure S2). The weak scattering of O with respect to heavy atoms such as Ce and La did not permit having reliable values on the sub-stoichiometry of the oxygen lattice, although a progressive decrease in the oxygen content is observed (upper inset of Figure 1C). The expansion of the ceria lattice and the formation of oxygen vacancies increase the lability and mobility of lattice oxygen.^19^ The samples present an average domain size of around 26 nm for the support, while the characteristic diffraction peaks of nickel are not detected due to the small crystalline domain size in Ni particles, related with the fast formation of this phase during auto-ignition of a viscous gel.^22^

Neutron diffraction experiments (NPD) were performed to identify the structural features of the fluorite matrix of composition Ce_0.9_La_0.1_O_2−δ_, concerning the presence of oxygen vacancies induced by the La^3+^ doping at the Ce^4+^ structural sites. The NPD diagram is perfectly indexed in the cubic *Fm*-3*m* space group; no impurities or additional reflections that could indicate departure from this symmetry were detected (Figure S3 and Table S2). As a second phase, metallic Ni was included in the refinement, defined in the space group *Fm*-3*m* with *a* = 3.5223 Å. Despite the relatively low amount present in the cermet (not visible from the XRD patterns), the large scattering factor of Ni for neutrons allowed us to confirm its presence as a metal. The formation of metallic nickel in the samples is corroborated by their characteristic dark gray color, by their magnetic properties, and also by HRTEM, as confirmed in a previous work.^22^ The most important conclusion of this NPD study is the refined value of the oxygen content of 1.96(1) per formula unit, which agrees perfectly, within the standard deviations, with the expected amount from the La doping level of *x* = 0.1.

The morphology of the prepared catalysts was further studied by scanning electron microscopy (SEM), which reveals a highly macroporous structure with an average pore size between 1 and 3 μm (Figure 1B). STEM micrograph and EDX analyses also show that samples exhibit a porous structure, with a uniform distribution of La into the ceria lattice (Figure 1B), while the nickel phase is relatively well dispersed over the support surface with sizes between 10 and 20 nm (Figure 1B and Figures S4 to S6). In a previous work,^22^ we performed an extensive study of the catalysts by TEM/HRTEM, determining the interplanar distance in the support (Ce_0.9_La_0.1_O_*x*_), having a value of 3.18 Å (d(111)), a little greater than that in bare CeO_2_. To confirm the doping by this technique, we have analyzed the sample Ni0.07/Ce0.8La0.2 (see Figure S7). The interplanar distance of the support reveals an increase in this parameter (3.26 Å), which is in accordance with the inclusion of a greater proportion of La in the ceria lattice.

The BET surface areas of some samples (determined by N_2_ adsorption–desorption isotherms) are listed in Table S3, varying between 10 and 20 m^2^/g for the Ni_*y*_(Ce_1–*x*_La_*x*_O_2–*x*/2_)_1–*y*_ samples. For the samples without Ni (CeO_2_ and Ce_0.9_La_0.1_O_1.95_), the BET surface areas are larger since the porous structure is not partially restricted by Ni particles. Microporosity was not found in any of the samples. The pore volume of mesopores calculated by this technique is negligible (0.001–0.003 cm^3^·g^–1^). The high macroporosity of these materials was confirmed by Hg porosimetry (see as an example the pore size distribution, average pore size, surface area, and porosity for the Ni0.07/Ce0.9La0.1 catalyst (Figure S8). This property is suitable for reactions at a very high space velocity, such as the ones carried out in this work.

First, we will comment on the influence of Ni loading on the catalytic behavior for the rWGS reaction (700 °C, H_2_/CO_2_ = 2). As expected, by thermodynamics, CO_2_ conversion decreases and CH_4_ selectivity increases with the decrease in reaction temperature (Table S4); for that reason, all reactions were performed at 700 °C.

The presence of nickel as an active phase leads to a dramatic improvement in CO_2_ conversion of more than 20% (at the beginning of the reaction) compared to the bare La-doped ceria support, as we have reported.^22^ These studies show an optimum Ni proportion around 7% molar (*y* = 0.07). After 6 h of time-on-stream, the system exhibits an average conversion of 57% (Figure S9), close to the equilibrium conversion under the same reaction conditions (59.3%) (thermodynamics data calculated by the authors), and a CO selectivity close to 99%, achieving the maximum CO yield in this reaction period. The catalysts suffer deactivation that is more pronounced when the Ni loading is considerably lower (*y* = 0.01 or 1% molar) than the optimum value (*y* = 0.07 or 7% molar), while higher Ni loadings (*y* = 0.1 or 10% molar) result in similar CO_2_ conversion and CO selectivity to the optimum loading. Although, as stated above, the Ni particles core is in the metallic state, the evolution of the CO selectivity with reaction time evidences an induction period that is related to the time needed to achieve the reduction of the available nickel phase, in close interaction with the support, to the metallic state.^5,34−37^ This induction period increases quite linearly with the proportion of nickel in each catalyst (inset in Figure S9b). For the catalyst without Ni (Ce_0.9_La_0.1_O_*x*_), the selectivity toward CO is 100% since the reaction beginning because Ni is active not only for the RWGS reaction but also for the methanation reaction.

**Figure 2:**
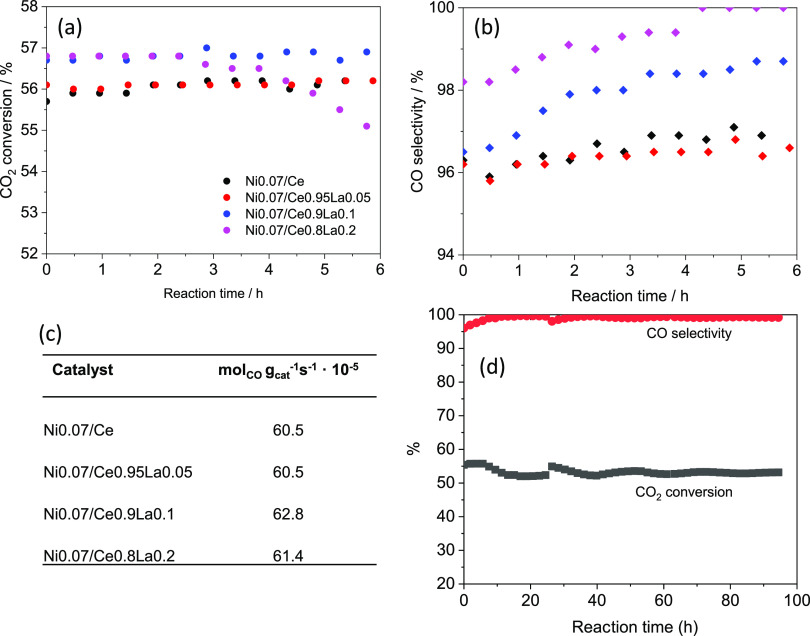
Catalytic performance of the Ni_0.07_/Ce_1–*x*_La_*x*_ series: (a) CO_2_ conversion and (b) CO yield for the rWGS reaction (700 °C, H_2_/CO_2_ = 2 (molar), 3 × 10^5^ mL_N_·h^–1^ g^–1^ of catalyst). (c) CO yield (mol_CO_·g_cat_^–1^·s^–1^), 6 h of time-on-stream and (d) durability test for the Ni0.07/Ce0.9La0.1 catalyst under 95 h.

The role of La doping was studied for the series of catalysts with different Ni loadings (see Figure S10, for the series with 4% of Ni). Figure 2 shows the results for the optimized nickel loading of 7% (the composition that produces the maximum CO yield after 6 h of reaction). The increase in La content leads to an improvement in CO_2_ conversion, which reaches an average of 57% for the Ni_0.07_/(Ce_0.9_La_0.1_O_1.95_)_0.93_ catalyst, indicating the significant role of oxygen vacancies in the rWGS performance. It is worth mentioning that the catalyst doped with 20% of La suffers deactivation (Ni0.07/Ce0.8La0.2). On the other hand, for lower La concentrations of 10% molar, the CO selectivity increases with reaction time due to the progressive reduction of the nickel phase,^14,32^ with methane being the only other product of the reaction.

To compare the CO yield among the activity of other RWGS catalysts, Table S5 depicts the yield expressed as mol CO produced·g catalyst^–1^·s^–1^, highlighting the reactivity of the optimized catalyst developed in this work.

The exceptional high CO formation (Figure 2c) shows the excellent activity of our samples for the rWGS reaction compared to the state-of-the-art catalyst (Table S5).^38^ One of the best yields of CO from the rWGS reaction that has been obtained under reaction conditions more similar to ours has been reported for Ni supported on Ce_0.75_Zr_0.25_O_2_, working at 700 °C, H_2_/CO_2_ = 3, and a space velocity of 120 L/h·g, showing the highest CO yield of 22.3 × 10^–5^ mol_CO_ g_cat_^–1^ s^–1^. Our study presents an optimized Ni0.07/Ce0.9La0.1 catalyst that has a CO yield (58 × 10^–5^ mol_CO_·g_cat_^–1^·s^–1^) that is 2.6 times higher, working at the same temperature of 700 °C but approximately 2.5 times higher space velocity (300 L/h·g) and at a lower H_2_/CO_2_ ratio (2), which means that in our case, the equilibrium is less shifted to the formation of products. Considering the CO_2_ conversion found for the catalyst Ni0.07/Ce0.9La0.1 (52%), after nearly 100 h of reaction, the TOF for CO_2_ conversion is 1.08 × 10^5^ h^–1^ (∼30 seg^–1^). For this calculation, we consider a Ni dispersion of ∼4.6%, with hemispherical active Ni particles and an average size of about 22 nm, as calculated by H_2_ pulse chemisorption.

The stability of our catalysts is also evidenced in the durability tests (Figure 2d), showing a uniform CO_2_ conversion above 52% and a CO selectivity higher than 99.6% during nearly 100 h of reaction, without deactivation.

**Figure 3:**
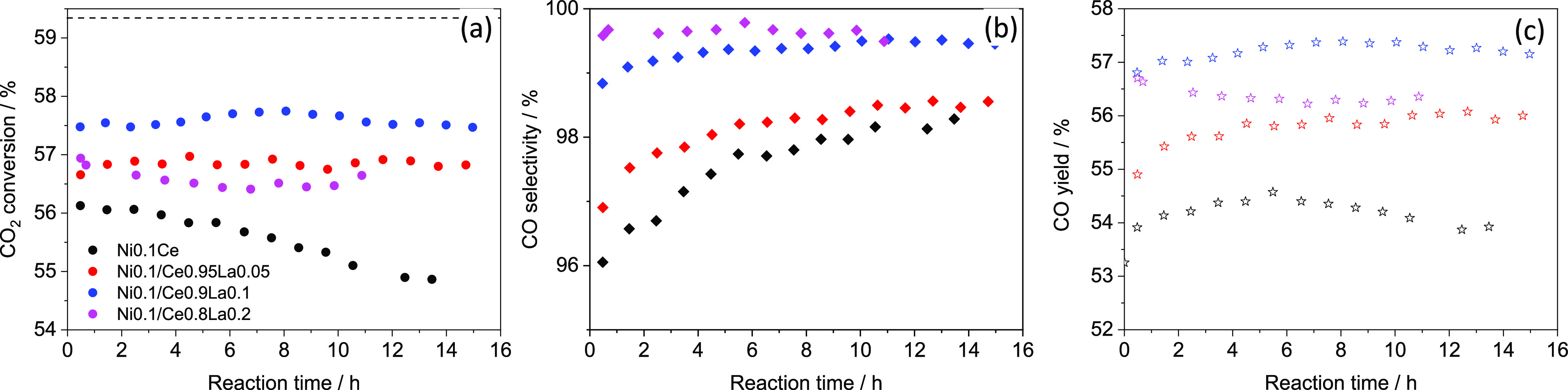
Catalytic performance of Ni_0.1_(Ce_1–*x*_La_*x*_O_2–*x*/2_)_0.9_ for the rWGS reaction (700 °C, H_2_/CO_2_ = 2 (molar), 6 × 10^5^ mL_N_·h^–1^ g^–1^ catalyst). (a) CO_2_ conversion vs reaction time, (b) CO selectivity vs reaction time, and (c) CO yield.

Finally, the remarkable efficiency of these La-doped catalysts is revealed by performing the catalytic tests even under high demand reaction conditions (double space velocity cf. 6 × 10^5^ mL_N_ h^–1^·g^–1^ of catalyst), where for Ni_0.1_/(Ce_0.9_La_0.1_O_1.95_)_0.9_, CO_2_ conversion and CO yield reach a maximum average value of 57.6% and 57.2%, respectively (Figure 3).

To obtain a better understanding of this remarkable catalytic behavior, a series of characterization techniques and computational studies have been performed. H_2_ reduction profiles were analyzed to gain insights into the influence of the La proportion on the redox properties of the catalysts. As an example, TPR-H_2_ experiments of Ni_0.07_/(Ce_1–*x*_La_*x*_O_2–*x*/2_)_0.93_ samples are described. Reduction profiles show the first intense and well-defined peak centered around 250 °C (Figure S11), which is related to the reduction of the NiO layer that passivates bulk metallic Ni particles. Since the inclusion of Ni into the support lattice has not been observed (see Table S2), as reflected by the results obtained by NPD, the H_2_ consumption peak cannot be related to the reduction of adsorbed oxygen species at oxygen vacancies that would occur during the formation of a Ce_*x*_Ni_*y*_O solid solution,^39^ confirming the formation of metallic nickel nanoparticles supported on La-doped ceria, which have been evidenced by an extensive study of these catalysts by transmission electron microscopy, recently reported by our group.^22^ Concerning the less intense and wider H_2_ consumption peak (Figure S11) in the range of 500–800 °C, it is ascribed to the reduction of NiO_*x*_ species that have a strong interaction with the support and also to the reduction of surface ceria.^21,34,35^ A progressive shift to lower reduction temperatures as La loading increases is observed in the H_2_-TPR profiles (Figure S11). This behavior is because ceria reduction is highly favored by the substitution of Ce^4+^ with an atom with higher atomic radius such as La^3+^, accompanied by the expansion of the lattice. This increases the lability and mobility of lattice oxygen.^40^ The decrease in the area of this peak with an increasing La loading is logical because a greater doping proportion is accompanied by a lower amount of ceria to be reduced.

To determine the role of ceria reduction and oxygen vacancies both in the bulk and on the surface of the catalysts, Raman spectroscopy measurements were performed in used catalysts and NAP-XPS analyses over the catalysts under reaction conditions were also carried out.

Before reporting and discussing these results, it is worth pointing out the origin and difference between extrinsic and intrinsic oxygen vacancies in the catalysts. La doping in CeO_2_ can induce O vacancies (extrinsic oxygen vacancies) via charge compensation as shown in the following processes described in Kroger–Vink notation:
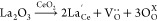


Additionally, the crystalline structure of La-doped CeO_2_ may accommodate more easily more oxygen vacancies that may favor the formation of oxygen vacancies (intrinsic oxygen vacancies) via reduction of Ce^4+^ to Ce^3+^ in the process described below:
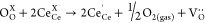


As previously commented, this is due to the larger unit cell of the Ce_1–*x*_La_*x*_ mixed oxide that favors oxygen lability and mobility under reduction conditions such as those used in the reaction feed.

The Raman spectra of all the samples (series Ni_*y*_/(Ce_1–*x*_La_*x*_O_2–*x*/2_)_1–*y*_ (*y* = 0.04 and 0.07) show a high-intensity band at 455 cm^–1^, related to the symmetrical stretching mode (νs(Ce-O)) of the CeO_2_ vibrational unit in the cubic fluorite lattice (Figure S12a). Meanwhile, the observed shoulder in the range 510–680 cm^–1^ includes two contributions attributed to the presence of lattice oxygen vacancies and reduced Ce^3+^ cations.^41−44^ On the one hand, it was assigned to extrinsic (nominal) oxygen vacancies produced to maintain the electroneutrality when Ce^4+^ ions are replaced by La^3+^ ions (α-band 561 cm^–1^). On the other hand, it is also related to the presence of intrinsic oxygen vacancies, generated by the presence of Ce^3+^ ions that resulted from oxygen removal (β-band 610 cm^–1^). As expected, extrinsic oxygen vacancies linearly increase with La concentration (Figure S12b).

Concerning the intrinsic oxygen vacancies, they increase with the proportion of La in the series with 4 and 7% Ni, as expected, since the larger size of the unit cell favors oxygen lability and mobility under the reductive conditions of the reaction feed. (Table S6). The correlation between CO yield versus the proportion of nominal oxygen vacancies (Figure S12c) exhibits a volcano plot with an optimum La concentration of 10%. Among other factors, oxygen pumping, associated with attainable Ce^3+^ formation (CeO_2_ ⇆ Ce_2_O_3_ + ^1^/_2_O_2_), may have an influence on promoting the rWGS pathway, favoring water formation.

**Figure 4:**
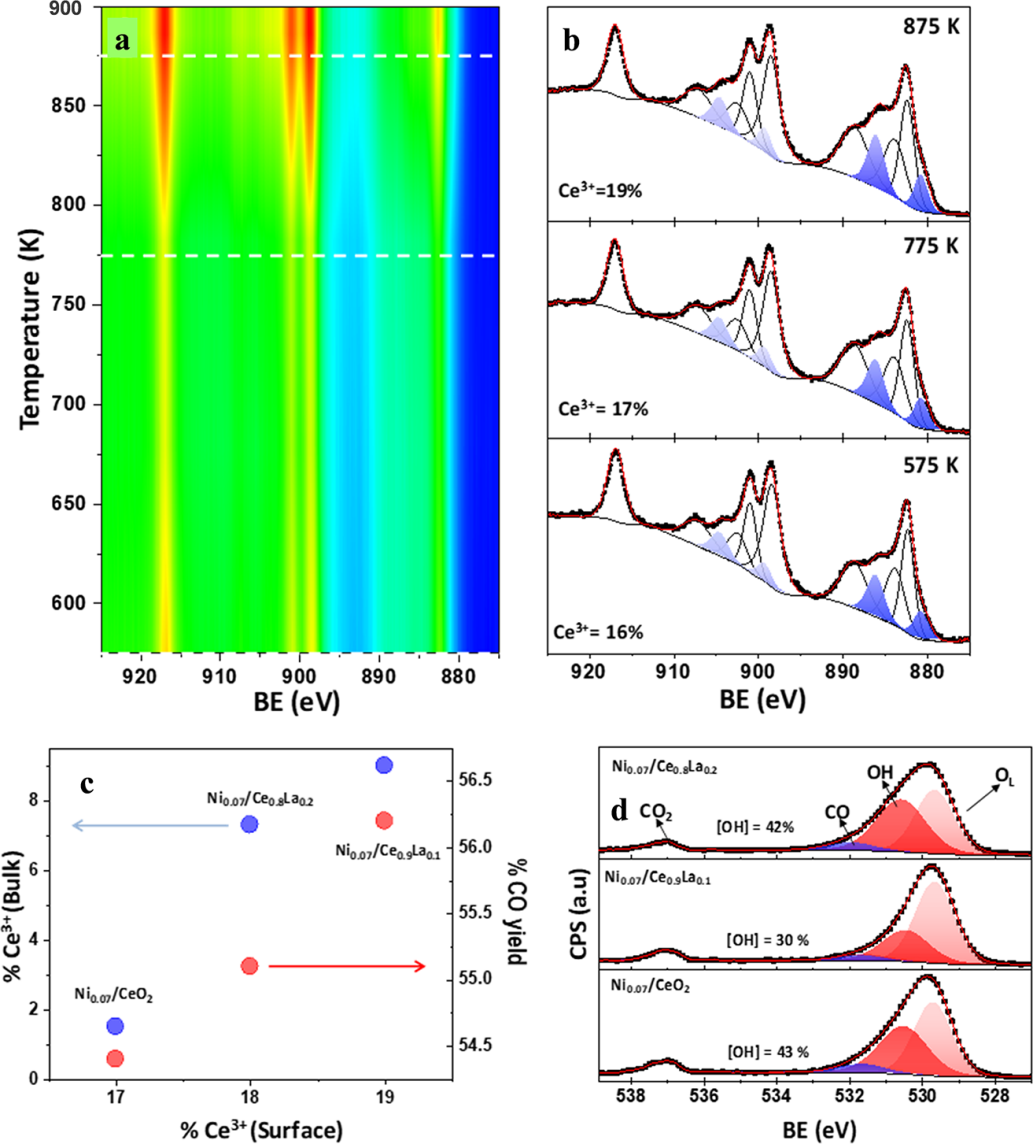
In situ characterization under reaction conditions for H_2_:CO_2_ = 2:1. (a) 2D and (b) selected variable temperature NAP-XPS of Ce 3d for sample Ni0.07/Ce0.9La0.1. (c) Correlation of surface (NAP-XPS) and bulk (Raman) Ce^3+^ concentration with CO yield (%) and (d) O 1s signal for different Ni_0.07_/Ce_1–*x*_La_*x*_ samples at 875 K.

On the other hand, surface characterization of Ni0.07/Ce, Ni0.07/Ce0.9La0.1 and Ni0.07/Ce0.8La0.2 samples was performed by NAP-XPS under a reactive mixture of H_2_ and CO_2_ in a 2:1 ratio at different temperatures. The analysis of the chemical state of Ni by XPS is difficult because the Ni 2p signal strongly overlaps with that of La 3d, but the Ce 3d and O 1s regions have been examined. Figure 4a depicts the changes in the Ce 3d region of the Ni0.07/Ce0.9La0.1 sample. The Ce^3+^ contribution becomes larger with increasing temperature (Figure 4b), a fact influenced by the reductive reaction feed (H_2_/CO_2_ = 2). At low temperatures, the increase in the Ce^3+^ contribution is significantly larger for both La-doped samples than for the undoped one (Figure S13 and Table S7), whereas at higher temperatures, maximum Ce^3+^ concentrations of 19% (Ce^3+^/Ce) and 16% (Ce^3+^/(Ce + La)) are obtained for the sample with 10% of La. As observed in Figure 4c, the CO yield also increases with the relative proportion of surface Ce^3+^ sites. The figure also highlights that the optimum La proportion in these rWGS catalysts is around 10% molar, which corresponds to the maximum amount of Ce^3+^ sites, accompanied by the maximum CO yield.

It is important to keep in mind that the bulk reduction of the support is favored with the increase in La (when it is incorporated in the unit cell of the fluorite, in the positions of Ce), but obviously, an increase in La doping implies a decrease in Ce, consistent with the fact that a maximum proportion of oxygen pump has an influence over the maximum proportion of Ce^3+^ on the surface.

The NAP-XPS experiment is performed under reductive conditions close to those of the reaction (with a gas mixture of H_2_ and CO_2_ (H_2_/CO_2_ = 2)). A lower surface concentration of Ce^3+^ than expected in the more reducible La-doped CeO_2_ systems under the reaction atmosphere suggests that the cerium oxide is involved in the CO_2_ activation and reaction mechanism.

As mentioned earlier in the Introduction, working at high temperatures and using Ni as the metallic phase, the formation of water, produced by the reaction of H atoms coming from the metal particles with the oxygen coming from the partially reduced ceria, seems to be the rate-limiting step for the rWGS reaction, and hydroxyl groups play an important role in water production (rWGS rate-determining step (RDS)). Their concentration was determined under reaction conditions by NAP-XPS, where the O 1s region (Figure 4d) shows three peaks assigned to lattice oxygen (O_L_, centered at 529.7 eV), hydroxyl species (OH, at 530.6 eV), and carboxyl species (CO, at 531.8 eV).^45,46^ It is clear that the Ni0.07/Ce0.9La0.1 sample, which shows a higher Ce^3+^ concentration, exhibits a significantly lower amount of OH species than other samples. Therefore, a lower proportion of adsorbed hydroxyl groups under reaction conditions suggests a more favored water formation and desorption, which correlates well with the higher catalytic activity observed for the 10% La-doped catalyst.

To evaluate a possible correlation between the surface basicity of the materials and their catalytic performance, we have performed CO_2_-TPD tests for the series Ni_*y*_/(Ce_1–*x*_La_*x*_O_2–*x*/2_)_1–*y*_ (*y* = 0.1). The CO_2_-TPD profiles of Ni0.1/Ce (Figure S14) showed three major desorption peaks located at 72, 439, and 660 °C, which are associated with weak, medium, and strong surface basic sites, respectively. According to the strength of surface interaction between CO_2_ and CeO_2_, these peaks can be attributed to the adsorption of monodentate carbonates, bidentate carbonates, and linearly adsorbed species for low (20–200 °C), medium (200–450 °C), and high (>450 °C) temperature desorption peaks, respectively.^47^

La-doped CeO_2_ catalysts only exhibited broad low-medium temperature peaks, mostly centered at ca. 69–110 °C. In particular, the medium temperature desorption peak was only evident for the sample with the lowest La content (Ni0.1/Ce0.95La0.05). Initially, this could suggest a loss of surface basicity upon La incorporation, if compared to the Ni/CeO_2_ sample. However, it has to be noticed that the La content may favor the formation of highly stable carbonates or lanthanum oxycarbonates,^48^ which remain adsorbed on the catalyst surface at temperatures of 800 °C (under the conditions used in these TPD experiments). This fact would explain the smaller CO_2_ desorption for the catalysts doped with La. The formation of these surface oxycarbonates may have a role in the gasification of carbon precursors.

The catalytic influence of the existence of nickel on the ceria surface has also been assessed by performing DFT calculations of the activation of CO_2_ and H_2_ on model Ni/ceria catalysts. To this end, previously reported models,^26,49−51^ consisting of four nickel atoms forming a bi-dimensional flat rhombohedral-shaped Ni_4_ cluster in direct contact with the oxidized and fully reduced ceria surfaces, were considered, hereinafter referred to as Ni_4_.CeO_2_ and Ni_4_.Ce_2_O_3_, respectively. With these model catalysts we do not only model a material with different degrees of reduction, but they also enable us to investigate the influence of the electronic perturbations on Ni species that are directly at the Ni–ceria interface; on CeO_2_, all four Ni atoms in direct contact with the support are oxidized (4 × Ni^0.5+^), whereas on Ce_2_O_3_, they are metallic (4 × Ni^0^).

**Figure 5:**
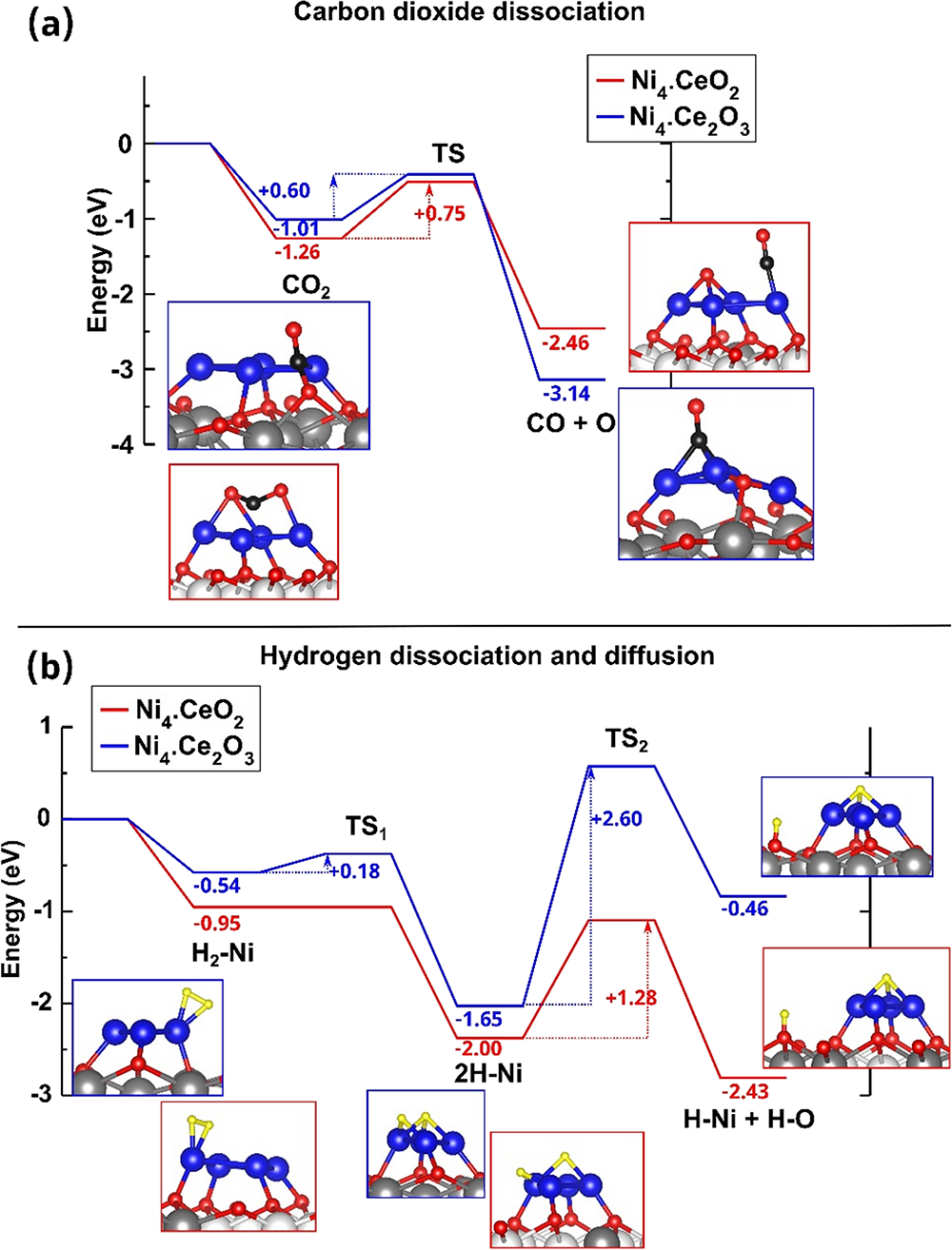
Reaction pathways for CO_2_ dissociation and H_2_ dissociation and diffusion on nickel-ceria-based catalysts. Reaction pathways for (a) CO_2_ dissociation on Ni_4_.CeO_2_ and Ni_4_.Ce_2_O_3_ and (b) hydrogen dissociation and diffusion on Ni_4_.CeO_2_ and Ni_4_.Ce_2_O_3_. The structures shown correspond to the side views of the optimized initial and final states used in the search of the transition state structure. All energies are relative to (a) CO_2_ and (b) H_2_ in the gas phase. Atom color code: nickel atoms are depicted in blue, oxygen in red, carbon in black, hydrogen in yellow, Ce^4+^ in white, and Ce^3+^ in gray.

Lu et al. reported that on the oxidized CeO_2_(111) surface, the CO_2_ dissociation reaction is highly endothermic by 3.23 eV with a high energy barrier of 3.70 eV.^52^ However, on the partially reduced CeO_2–*x*_(111) surface, they showed that the dissociation process is exothermic by 0.52 eV with no activation barrier,^52^ in line with previous studies.^53−55^ On the surface of the Ni_4_.CeO_2_ and Ni_4_.Ce_2_O_3_ model catalysts, the CO_2_ dissociation is exothermic by 1.20 and 2.13 eV, respectively, with energy barriers of 0.75 and 0.60 eV, respectively (see Figure 5a, Figure S15, and Table S8). We noted that Zhang et al. have recently reported a CO_2_ activation barrier of 1.6 eV on Ni_4_.CeO_2_,^51^ which is higher by 0.85 eV than the one we find (Figure 5a). We further noticed that the reaction paths are not the same because the final states are not identical, that is, in our case, the reaction energy is exothermic by 1.2 eV, whereas that reported by Zhang et al. is endothermic by 0.39 eV.^51^ We point out that the Ni_4_.CeO_2_ surface exhibits a reactivity toward CO_2_ much higher than those of the perfect CeO_2_(111) surface, for which the results^52^ indicate the thermodynamic stability of inert CO_2_, and on the extended Ni(111) surface, for which CO_2_ does not even bind (Figure S16 and Table S8), contrary to Ni_4_.CeO_2_ and Ni_4_.Ce_2_O_3_.

The electronic structure and structural perturbations of small particles of Ni in contact with ceria^26,27^ affect the reactivity of the supported particles. If there are not oxygen vacancies at the ceria support (CeO_2_), the CO_2_ dissociation takes place on the partially oxidized Ni nanoparticle (4 × Ni^0.5+^), whereas if there are vacancies (as it is the case of Ce_2_O_3_), the dissociation takes place cooperatively at the interface between the metallic Ni (4 × Ni^0^) particle and the support (Figure 5a); expectedly, a path that does not involve the reduced support has a substantially higher activation barrier (by 0.67 eV, Figure S15). Undoubtedly, oxygen vacancies on the ceria support are crucial for CO_2_ activation and decomposition into CO and O on Ni/ceria catalysts, according to the abovementioned absence of activation barrier over a surface vacancy on the CeO_2_(111) surface. The rWGS also requires the activation of the H_2_ molecule. Fernández-Torre et al. previously showed that the dissociation of H_2_ on the oxidized ceria surface requires to overcome an activation barrier of 1 eV.^56^ On Ni(111), H_2_ occurs with a small energy barrier of 0.08 eV, and the reaction is exothermic by 1.05 eV with a barely bound molecular precursor (see Table S8 and Figure S16). However, on the ceria-supported small Ni particles, the activation barriers are also very low (0.00–0.18 eV) (Table S8 and Figure 5b), but the binding of both the initial and H + H final state is stronger by up to about 1 eV compared to Ni(111), which indicates that Ni nanoparticles in Ni/ceria catalysts are essential for the activation of H_2_.

The influence of the degree of reduction of the ceria support on the barrier for the migration of H atoms from the Ni center to oxygen atoms of the support has also been assessed by DFT (Figure 5b). If the support is fully reduced (Ce_2_O_3_), the reaction is endothermic by 1.19 eV with a diffusion barrier of 2.60 eV, which corresponds to twice the value (1.28 eV) for the support without oxygen vacancies (CeO_2_). In the latter case, H diffusion is exothermic by 0.43 eV. This result indicates that the more oxygen vacancies the support has, which happens if we dope more and more with La, the diffusion and formation of OH, the step prior to the formation of H_2_O, becomes more and more difficult.

The kinetics of the CO_2_ reduction with H_2_ was investigated for the Ni0.07/Ce0.9La0.1 catalyst in the temperature range of 700–775 °C.

The apparent activation energy obtained for this catalyst for the rWGS reaction via an Arrhenius-type function was 26 kJ·mol^–1^, and the rate equation is shown here below (eq 1):
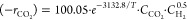
1

*r*_CO_2__ = *k*·[CO_2_]^α^·[H_2_]^β^ = *A*_0_·exp[−*E*_a_/*RT*]·[CO_2_]^α^·[H_2_]^β^ (*r*_CO_2__: mol CO_2_ converted/s·g of catalyst, *C*_CO_2__ or [CO_2_] and *C*_H_2__ or [H_2_]: moles/L, A_0_: moles CO_2_ converted/g of catalyst·s)·(L/mol)^α+β^, and *R*: 8.314472 J/mol·K).

**Figure 6:**
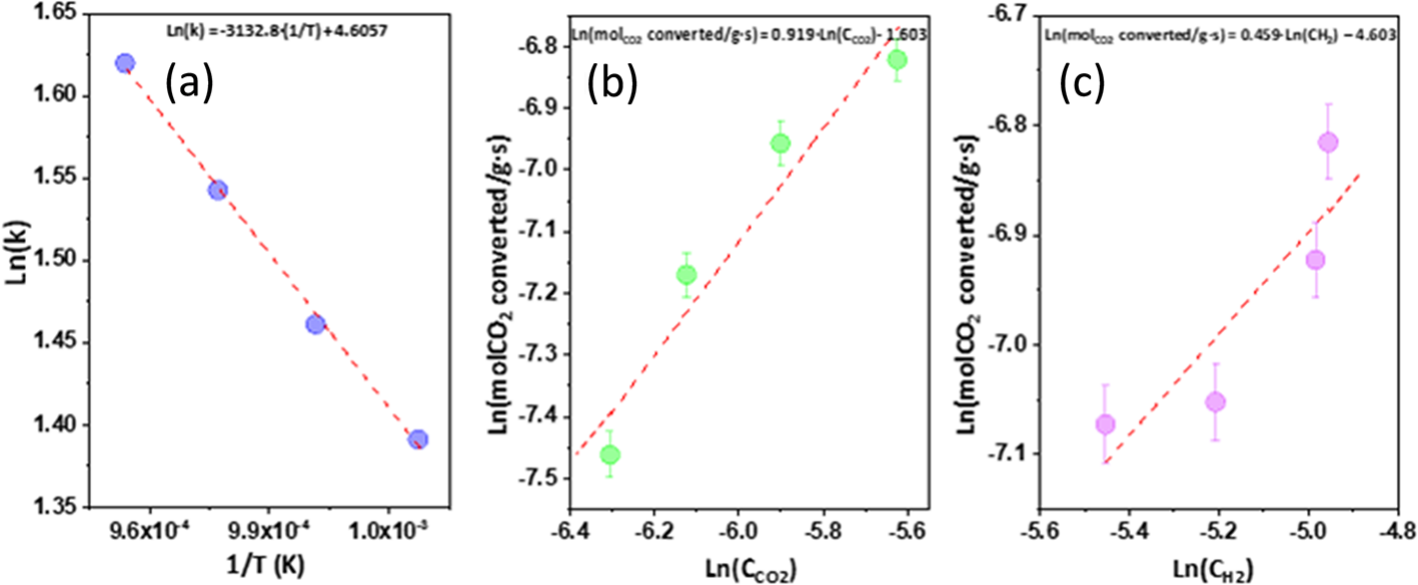
Kinetic study of the rWGS reaction at high temperatures for the Ni0.07/Ce0.9La0.1 catalyst. Kinetic plots for the determination of (a) the activation energy and the reaction orders with respect to (b) CO_2_ and (c) H_2_.

As far as we know, our kinetic study for the rWGS reaction is the first study performed using Ni/La-doped ceria at high reaction temperatures (700–775 °C). The calculated activation energy is close to those reported in the literature for the same reaction using a catalyst based on Cu/ZnO/Al_2_O_3_^57^ or Au supported on a reducible support (TiO_2_)^58^ but smaller than that obtained for a supported catalyst over a nonreducible support, such as alumina, which highlights the involvement of the support in the reaction. Thus, using a non-noble supported catalyst, we achieve a very fast rWGS reaction. On the other hand, the reaction orders for CO_2_ and H_2_ (Figure 6b,c, respectively) are 1 and 0.5, respectively, indicating that the H_2_ concentration has a lesser effect on the reaction rate, while the higher order obtained for CO_2_ implies a higher selectivity toward CO. These values agree with those reported in the literature for the homogeneous rWGS reaction, working at atmospheric pressure and high temperatures (at least 750 °C).^59,60^ The kinetic study performed by Wolf et al.^61^ for the rWGS reaction, also working at high temperatures using a Ni-Al_2_O_3_ commercial catalyst, lead to reaction orders of 1 and 0.3 for CO_2_ and H_2_, respectively. These values are close to the reaction orders obtained in our study, wherein for this Ni catalyst supported on alumina, the influence of hydrogen on the reaction rate is even lower than that in the present study.

In summary, the activity and selectivity of Ni_*y*_(Ce_1–*x*_La_*x*_O_2–*x*/2_)_1–*y*_ catalysts are influenced by the Ni loading and the La dopant proportion. Optimum values of these two variables in the catalyst composition would lead to very active and stable catalysts, with CO_2_ conversion close to the thermodynamic equilibrium (59.3% for these reaction conditions: 700 °C, H_2_/CO_2_(molar) = 2) and selectivity to CO > 99.5%, operating at a very high space velocity.

## 4 Conclusions

The elucidation of the structure and composition of catalysts for CO_2_ conversion is a major challenge in the development of highly efficient rWGS catalysts. To solve this, we propose the use of Ni-(La-doped CeO_2_)-based catalysts, less expensive than noble metal-based ones, prepared by a patented combustion method, with optimum values of Ni loading and dopant proportion, namely, 7 and 10% (molar), respectively. The system achieved an unprecedented average conversion of 52% working (after 96 h of time-on-stream) at a very high space velocity (300 L·h^–1^·g^–1^), which is very close to the equilibrium conversion (59.3%), and 100% selectivity to CO (58 × 10^–5^ mol_CO_·g_cat_^–1^·s^–1^). Experimental results and theoretical calculations reveal that the function of the metallic phase is that of activating H_2_ dissociation and promoting hydrogen spillover onto the catalyst support to produce H_2_O by removal of oxygen from the metal oxide lattice, which is accompanied by the formation of Ce^3+^ cations. The function of the reducible support is twofold, namely, to provide surface oxygen vacancies, where the dissociative adsorption of CO_2_ is activated, and to modulate oxygen mobility, which is modified by the incorporation of La in the lattice. The optimum value of 10% for the La concentration corresponds to a maximum surface proportion of Ce^3+^ and the highest CO yield. The joint action of the active sites determines the conversion and selectivity as a function of the catalyst composition. This fundamental understanding of both the structure–reactivity relationships and the nature of the active sites is an essential step toward the design of active, selective, and durable catalysts for rWGS, among other processes in which the activation of H_2_ and CO_2_ are involved.

## Supporting Information Available

Additional characterization details: NPD data, BET data, activity parameters as a function of reaction temperature, catalytic performance of representative traditional catalyst formulations, including our catalyst, Raman spectroscopy data, NAP-XPS data, energy profile for the dissociation of CO_2_ and H_2_ on nickel-ceria-based catalysts, XRD patterns, Rietveld fits of XRD patterns, NPD, STEM micrographs, EDX line profile, and EDX mapping, HRTEM micrographs, pore size distribution and textural data by Hg intrusion porosimetry, catalytic performance of Ni_*y*_/(Ce_0.9_La_0.1_O_1.95_)_1–*y*_ for the rWGS reaction, catalytic performance of Ni_0.04_(Ce_1–*x*_La_*x*_O_2–*x*/2_)_0.96_ series for the rWGS reaction, temperature reduction profiles of freshly prepared Ni_0.07_/(Ce_1–*x*_La_*x*_O_2–*x*/2_)_0.93_, CO_2_-TPD profiles, cooperative and non-cooperative reaction pathways for CO_2_ dissociation on Ni_4_.Ce_2_O_3_, and reaction pathways for CO_2_ and H_2_ dissociation on Ni(111) (PDF)

## Notes

DFT data supporting the findings of this manuscript are available in the open Materials Cloud repository (https://www.materialscloud.org/home) with the identifier: 10.24435/materialscloud:dc-46.
