# Supplementary material for: Highly Active and Stable Ni/La-Doped Ceria Material for Catalytic CO2 Reduction by Reverse Water-Gas Shift Reaction
Source: ACS Appl Mater Interfaces. 2022 Nov 2;14(45):50739–50. doi: 10.1021/acsami.2c11248 (PMC9673058; doi:10.1021/acsami.2c11248)
Supplement: Supplementary file 1 — am2c11248_si_001.pdf [file am2c11248_si_001.pdf]

# Supporting Information

# Highly Active and Stable Ni/La-Doped Ceria Material for catalytic $CO_2$ reduction by Reverse Water-Gas Shift Reaction

*Consuelo Alvarez-Galvan[1]*, Pablo G. Lustemberg[1,2], Freddy E. Oropeza[3], Belén Bachiller-Baeza[1], Martin Dapena Ospina[1], María Herranz[1], Jesús Cebollada[1], Laura Collado[3], José M. Campos-Martin[1], Víctor A. de la Peña-O'Shea[3], José A. Alonso[4] and M. Verónica Ganduglia-Pirovano[1,]***

[1] Instituto de Catálisis y Petroleoquímica (CSIC), Cantoblanco, 28049 Madrid, Spain

[2] Instituto de Física Rosario (IFIR), CONICET-UNR, 2000EZP Rosario, Santa Fe, Argentina

[3] Photoactivated Processes Unit, IMDEA Energy Institute, Avda. Ramón de la Sagra 3, 28935, Móstoles, Madrid, Spain

[4] Instituto de Ciencia de Materiales de Madrid (CSIC), Cantoblanco, 28049 Madrid, Spain

* Corresponding author: c.alvarez@icp.csic.es

** Corresponding author: vgp@icp.csic.es

**Table S1.** Nomenclature of $Ni_y/(Ce_{1-x}La_xO_{2-x/2})_{1-y}$ samples.

| **Catalyst** | **Nomenclature** |
|---|---|
| $Ni_{0.01}/(Ce_{0.9}La_{0.1}O_{1.95})_{0.99}$ | Ni0.01/Ce0.9La0.1 |
| $Ni_{0.04}/(CeO_2)_{0.96}$ | Ni0.04/Ce |
| $Ni_{0.04}/(Ce_{0.95}La_{0.05}O_{1.975})_{0.96}$ | Ni0.04/Ce0.95La0.05 |
| $Ni_{0.04}/(Ce_{0.9}La_{0.1}O_{1.95})_{0.96}$ | Ni0.04/Ce0.9La0.1 |
| $Ni_{0.04}/(Ce_{0.8}La_{0.2}O_{1.9})_{0.96}$ | Ni0.04/Ce0.8La0.2 |
| $Ni_{0.07}/(CeO_2)_{0.93}$ | Ni0.07/Ce |
| $Ni_{0.07}/(Ce_{0.95}La_{0.05}O_{1.975})_{0.93}$ | Ni0.07/Ce0.95La0.05 |
| $Ni_{0.07}/(Ce_{0.9}La_{0.1}O_{1.95})_{0.93}$ | Ni0.07/Ce0.9La0.1 |
| $Ni_{0.07}/(Ce_{0.8}La_{0.2}O_{1.9})_{0.93}$ | Ni0.07/Ce0.8La0.2 |
| $Ni_{0.1}/(CeO_2)_{0.9}$ | Ni0.1/Ce |
| $Ni_{0.1}/(Ce_{0.95}La_{0.05}O_{1.975})_{0.9}$ | Ni0.1/Ce0.95La0.05 |
| $Ni_{0.1}/(Ce_{0.9}La_{0.1}O_{1.95})_{0.9}$ | Ni0.1/Ce0.9La0.1 |
| $Ni_{0.1}/(Ce_{0.8}La_{0.2}O_{1.9})_{0.9}$ | Ni0.1/Ce0.8La0.2 |

**Table S2.** Atomic parameters of $Ni_{0.1}/Ce_{0.9}La_{0.1}$ refined from NPD data at 295 K.

| | ***x*** | ***y*** | ***z*** | $U_{iso}$ | **Occ.** |
|---|---|---|---|---|---|
| **Ce** | 0.00000 | 0.00000 | 0.00000 | 0.0084 (4) | 0.8995(6) |
| **Ni** | 0.00000 | 0.00000 | 0.00000 | 0.0084 (4) | 0.0005(6) |
| **La** | 0.00000 | 0.00000 | 0.00000 | 0.0084 (4) | 0.1 |
| **O** | 0.25000 | 0.25000 | 0.25000 | 0.0133 (3) | 0.975(5) |

Space group Fm-3m, a = 5.44438 (8) Å. RBragg= 1.77%; χ2= 1.55.

We have built a crystallographic model where some Ni is statistically distributed at the Ce positions (2a sites at the Fm-3m space group). The refinement yields a value of 0.00005(6) as occupancy factor of Ni, which is not significant within the standard deviation, with the same order of magnitude. Thus, we can conclude that there is no experimental evidence that Ni is introduced into the crystal structure of the fluorite matrix in the fresh prepared sample.

**Table S3.** BET surface area of catalysts

| Sample | BET Surface area ($m^2g^{-1}$) |
|---|---|
| $CeO_2$ | 33.1 |
| $Ce_{0.9}La_{0.1}O_2$ | 46.1 |
| $Ni_{0.04}/(CeO_2)_{0.96}$ | 10.4 |
| $Ni_{0.04}/(Ce_{0.95}La_{0.05}O_{1.975})_{0.96}$ | 15.5 |
| $Ni_{0.07}/(Ce_{0.9}La_{0.1}O_{1.95})_{0.93}$ | 19.7 |
| $Ni_{0.1}/(Ce_{0.95}La_{0.05}O_{1.975})_{0.9}$ | 15.4 |
| $Ni_{0.1}/(Ce_{0.9}La_{0.1}O_{1.95})_{0.9}$ | 17.7 |
| $Ni_{0.1}/(Ce_{0.8}La_{0.2}O_{1.9})_{0.9}$ | 11.8 |

**Table S4.** $CO_2$ conversion, CO and $CH_4$ selectivities for Ni0.07/Ce0.95La0.05 ($H_2/CO_2$ = 2 (molar); $3\cdot10^5$ $cm^3N\cdot h^{-1}\cdot g$ $catalyst^{-1}$) as a function of reaction temperature

| Reaction temperature (ºC) | $CO_2$ Conversion (%) | CO Selectivity (%) | $CH_4$ Selectivity (%) |
|---|---|---|---|
| 700 | 56.1 | 96.5 | 3.5 |
| 700 | 56.2 | 96.8 | 3.2 |
| 700 | 56.2 | 96.4 | 3.6 |
| 600 | 45.3 | 91.8 | 8.2 |
| 600 | 45.4 | 92.0 | 8.0 |
| 600 | 45.5 | 92.3 | 7.7 |
| 500 | 26.2 | 78.1 | 21.9 |
| 500 | 25.7 | 78.5 | 21.5 |
| 500 | 25.9 | 79.3 | 20.7 |

**Table S5**. Catalytic performance of representative traditional catalyst formulations, including our catalyst

| **Catalyst** | **T (°C)** | **$H_2/CO_2$ (mol)** | **Space velocity ($L\cdot h^{-1}\cdot g^{-1}$)** | **$\%CO_2$ conv./ (%CO Sel.)** | **$CO_2$ conv. (eq.)** | **mol CO/ $g_{cat}^{-1}s^{-1}\cdot 10^{-5}$ *** | **Ref.** |
|---|---|---|---|---|---|---|---|
| 1%Pt/$TiO_2$ | 600 | 1.5 | 12 | 56/(100) | 45 | 1.56 | 1 |
| (5.6%) Cu/$CeO_2$ nanosph. | 500 | 3 | 300 | 41/(100) | 49.5 | 33.5 | 2 |
| (1%) Ni-$CeO_2$(cp) | 700 | 1 | 12 | 28/(80) | 30.6 | 31.2 | 3 |
| (0.5 %) Ni/$Ce_{0.75}Zr_{0.25}O_2$ | 700 | 3 | 120 | 62.5/(99.5) | 67.8 | 22.3 | 4 |
| $Ni_{0.07}$/ $(Ce_{0.9}La_{0.1}O_{1.95})_{0.93}$ | 700 | 2 | 300 | 57/(99) | 59.3 | 58.0 | This work |

* data calculated considering the data reported in some articles

**Table S6**. Relative intensities of Raman bands at 560 $cm^{-1}$ (extrinsic oxygen vacancies) and 610 $cm^{-1}$ (intrinsic oxygen vacancies) in relation to the main Raman band of Ce–O in $CeO_2$ at 455 $cm^{-1}$ for the spent catalysts of series Ni0.04/$Ce_{1-x}La_x$ and Ni0.07/$Ce_{1-x}La_x$

| **Catalyst** | **α (560 $cm^{-1}$)** | **β (610 $cm^{-1}$)** |
|---|---|---|
| Ni0.04/Ce | 5.0 | 3.8 |
| Ni0.04/Ce0.95La0.05 | 6.9 | 5.1 |
| Ni0.04/Ce0.9La0.1 | 10.9 | 7.2 |
| Ni0.04/Ce0.8La0.2 | 20.6 | 11.8 |
| Ni0.07/Ce | 8.0 | 6.0 |
| Ni0.07/Ce0.95La0.05 | 10.1 | 7.6 |
| Ni0.07/Ce0.9La0.1 | 12.9 | 9.0 |
| Ni0.07/Ce0.8La0.2 | 24.6 | 14.3 |

The data reported in this table have been obtained normalizing the Raman band of the symmetric Ce-O vibrational mode at 455 $cm^{-1}$ (100%), and calculating the relative the intensity of the bands at 560 and 610 $cm^{-1}$.

**Table S7.** Surface atomic proportion of $Ce^{3+}$ determined by NAP-XPS under reaction at different temperatures ($H_2/CO_2 = 2$, molar).

| Catalyst | 575 K | 675 K | 775 K | 875 K |
|---|---|---|---|---|
| | | $Ce^{3+}/Ce^{4+}$ | | |
| Ni0.07/Ce | 12 | 15 | 15 | 17 |
| Ni0.07/Ce0.9La0.1 | 16 | 16 | 17 | 19 |
| Ni0.07/Ce0.8La0.2 | 16 | 16 | 15 | 18 |
| | | $Ce^{3+}/(Ce + La)$ | | |
| Ni0.07/Ce | 12 | 15 | 15 | 17 |
| Ni0.07/Ce0.9La0.1 | 14 | 14 | 15 | 16 |
| Ni0.07/Ce0.8La0.2 | 12 | 12 | 12 | 14 |

**Table S8.** Energy profile for the dissociation of $CO_2$ and $H_2$ on nickel-ceria based catalysts.

| Catalyst | IS | TS | FS | $E_{Barrier}$ |
|---|---|---|---|---|
| | | **$CO_2 \rightarrow CO + O$** | | |
| **Ni(111)** | 0.25 | 0.87 | -0.81 | 0.62 (0.52[5]) |
| **Ni.$CeO_2$** | -1.26 | -0.51 | -2.46 | 0.75 |
| **Ni.$Ce_2O_3$** | -1.01 | -0.41 | -3.14 | 0.60 |
| **$CeO_2$(111)[6]** | -0.38 | 4.08 | 2.85 | 3.70 |
| **$CeO_{2-x}$(111)[6]** | -0.69 | -0.69 | -1.21 | 0.00 |
| | | **$H_2 \rightarrow H + H$** | | |
| **Ni(111)** | -0.01 | 0.07 | -1.06 | 0.08 (0.04[7]) |
| **Ni.$CeO_2$** | -0.95 | -0.95 | -2.00 | 0.00 |
| **Ni.$Ce_2O_3$** | -0.54 | -0.36 | -1.65 | 0.18 |
| **$CeO_2$(111)[8]** | -0.03 | 0.97 | -1.15 | 1.00 |

Energy values (in eV) of the initial state (IS), final state (FS) and transition state (TS) of the reaction pathway for $CO_2$ and $H_2$ dissociation on Ni(111), $Ni_4.CeO_2$, and $Ni_4.Ce_2O_3$ surfaces. The activation barrier ($E_{Barrier}$) is also listed. The values in parentheses indicate activation barrier values reported in the literature.

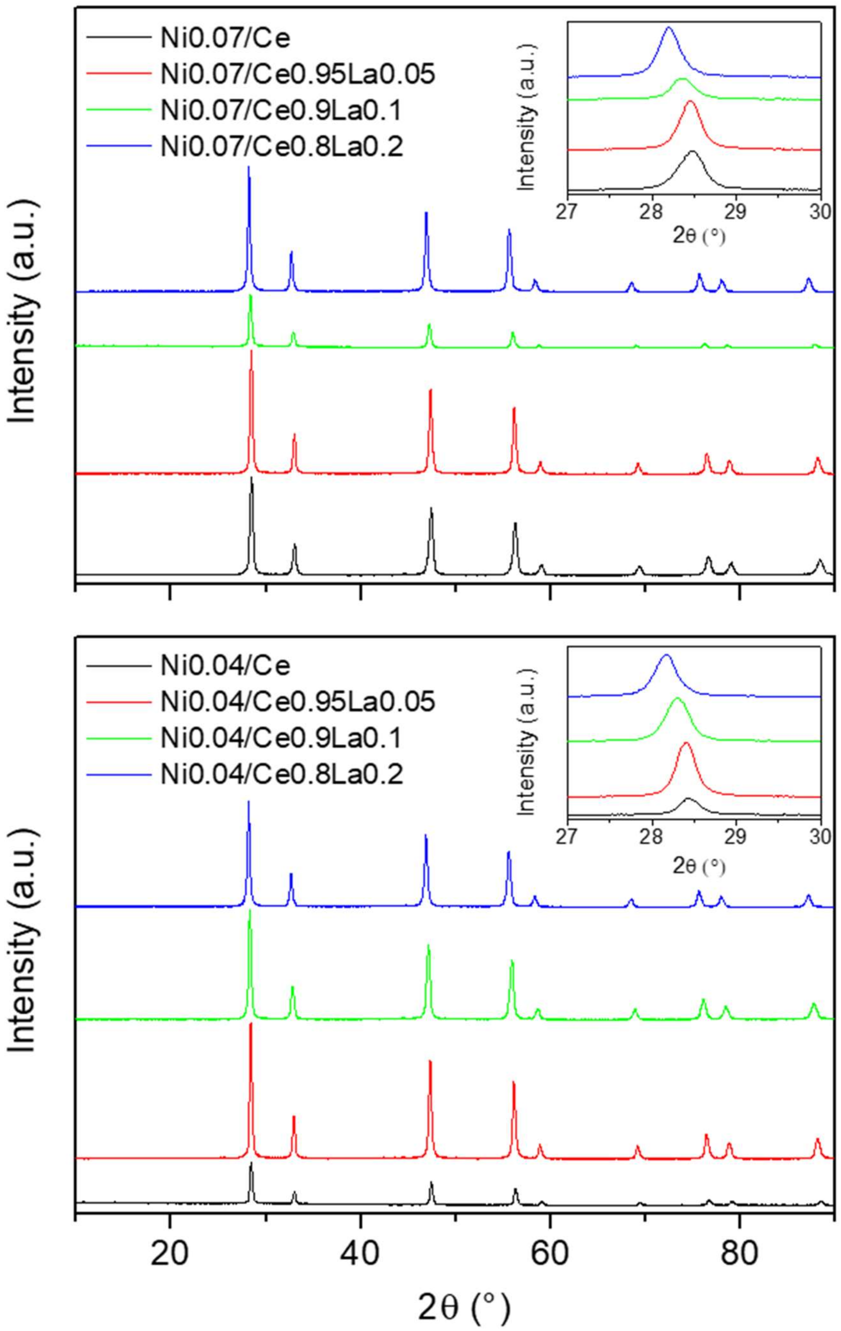


**Figure S1.** XRD patterns for $Ni_y/(Ce_{1-x}La_xO_{2-x/2})_{1-y}$, (x=0, 0.05, 0.1, 0.2; y = 0.04, 0.07) catalysts. (inset: expanded view)

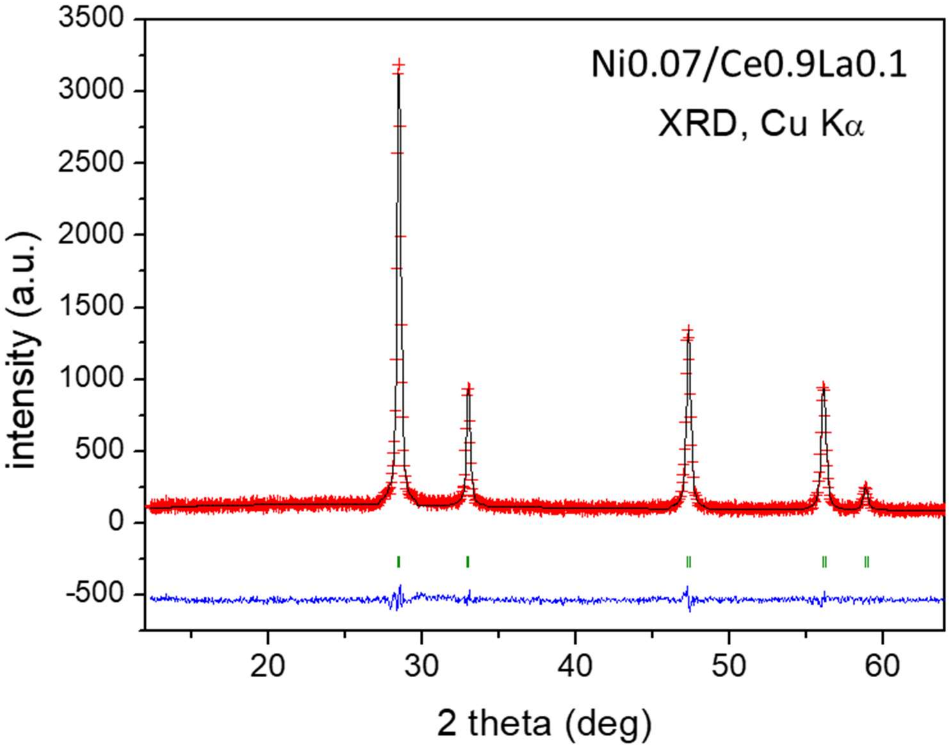


**Figure S2.** Rietveld fit of the XRD pattern of the sample Ni0.07/Ce0.9La0.1.

The pattern shows intense and sharp diffraction lines at angles 2θ of 28.6°, 33.1°, 47.4° and 56.4° corresponding to the reflection planes (111), (200), (220) and (311) of fluorite-structured $Ce_{0.9}La_{0.1}O_{1.95}$; the diffraction lines corresponding to metallic nickel cannot be distinguished. The crystal structure of the fluorite phase was defined in the cubic Fd-3m (No 225) space group, Z= 4. Ce and La atoms are distributed at random at 4a (0,0,0), and oxygen atoms O at 8c (1/4,1/4,1/4) sites. Although, the cermet is a composite of fluorite and metal Ni, given the relatively small amount of Ni (7% molar, 2.5% weight) and the small crystalline domain in Ni particles, this phase is not visible in the XRD diagram.

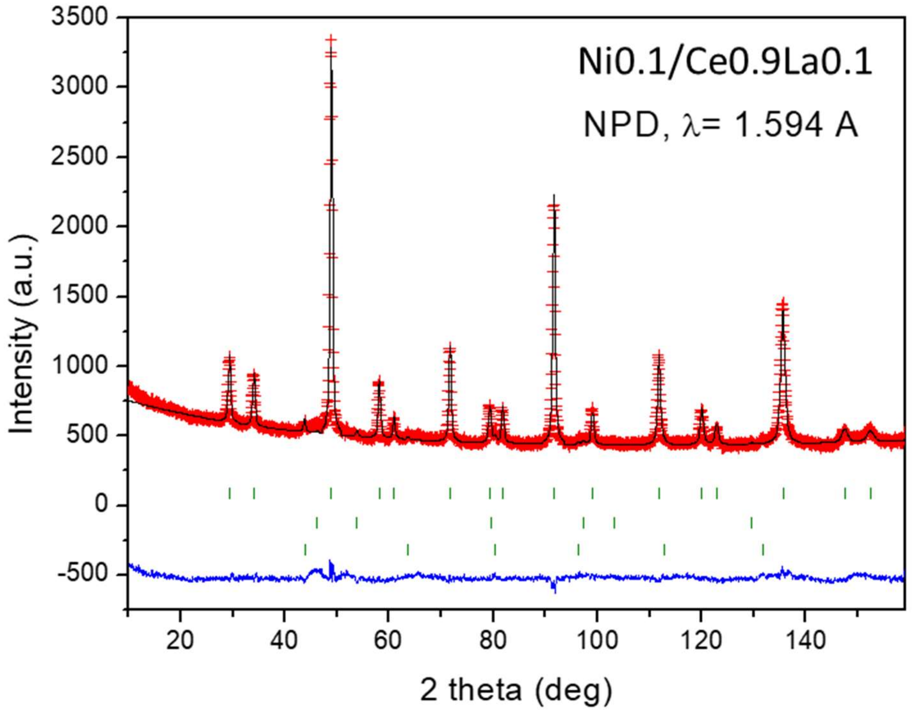


**Figure S3**. Neutron powder diffraction pattern for fresh Ni0.1/Ce0.9La0.1.

Our neutron data allowed us the access to a wide region of the reciprocal space enabling the successful refinement of the occupancy factors for O atoms, minimizing the correlation with the displacement factors. As a third phase, it was necessary to include V from the sample holder, defined in Im-3m with a= 3.0253 Å.

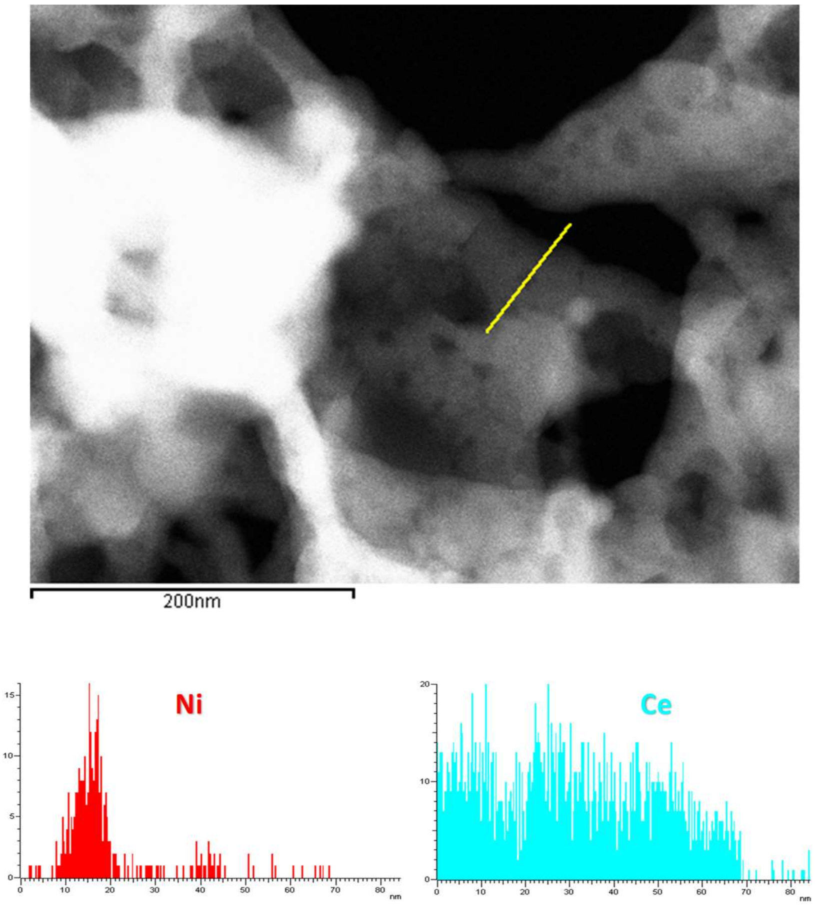


**Figure S4**. STEM micrograph of Ni0.04/Ce0.9La0.1 catalyst (after reaction) and EDX line profile on the yellow line.

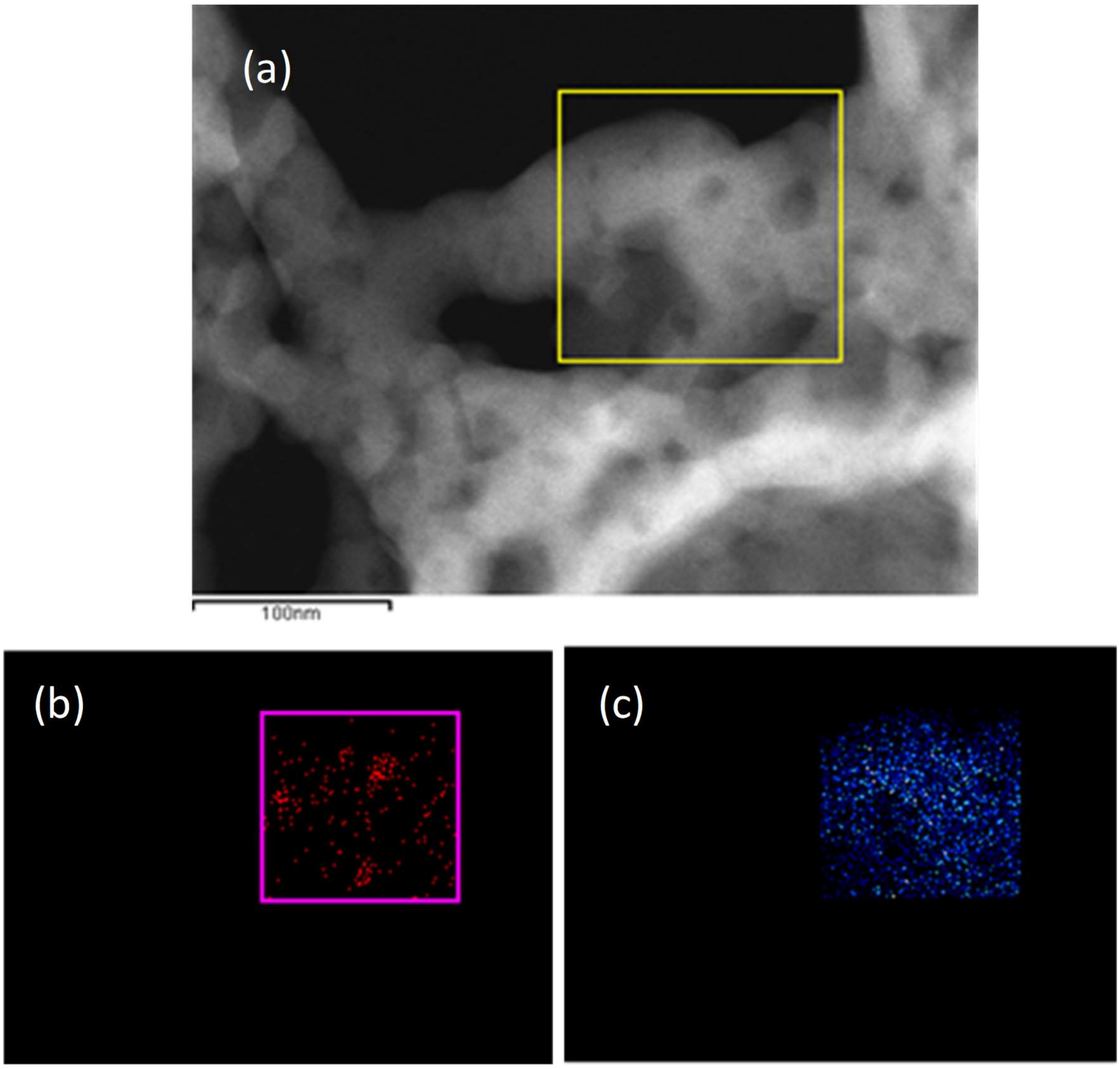


**Figure. S5.** STEM micrograph of the Ni0.04/Ce catalyst (after reaction) (a) and EDX mapping (b) Ni and (c) Ce.

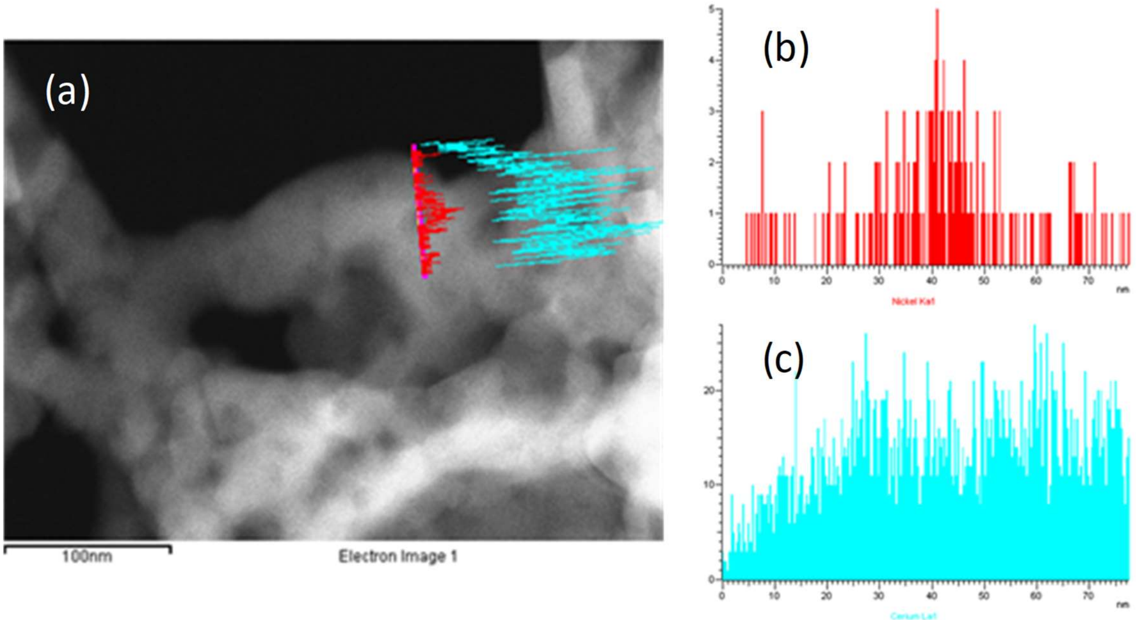


**Figure S6. (**a) STEM micrograph of Ni0.04/Ce catalyst (after reaction) and EDX line profile (b) Ni (c) Ce.

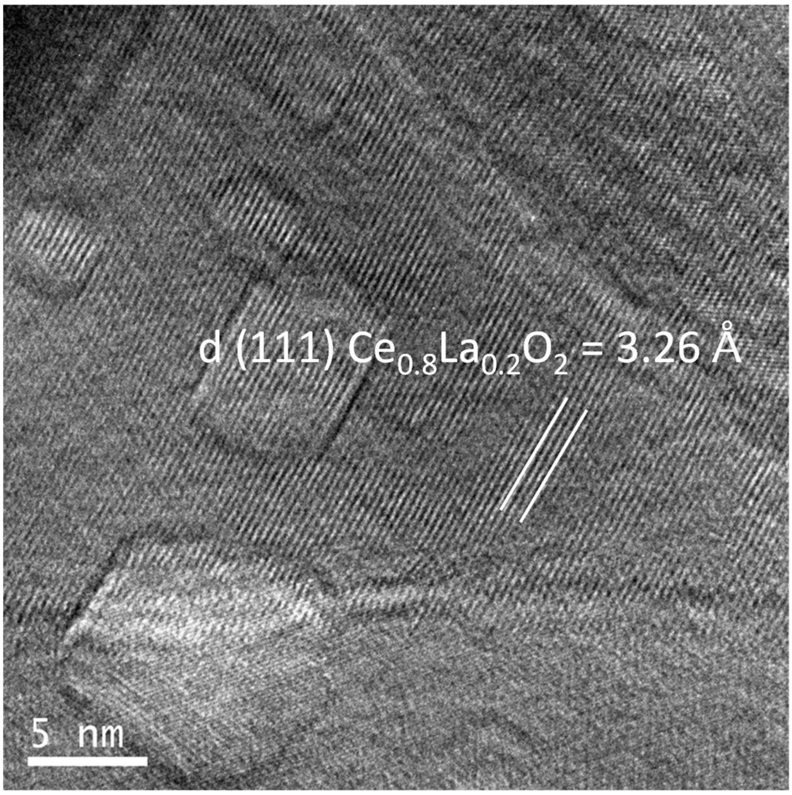


**Figure S7.** HRTEM representative micrograph for $Ni_{0.07}/Ce_{0.9}La_{0.1}$ fresh prepared catalyst

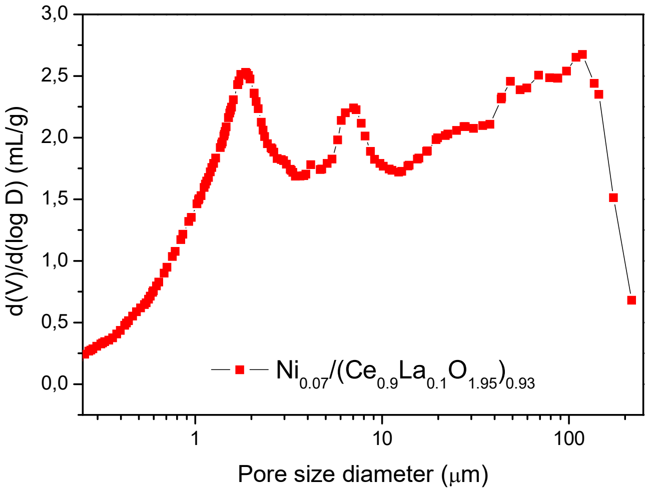


| | |
|---|---|
| Total Pore Area (m²/g) | 18.8 |
| Average Pore Diameter (4V/A) (μm) | 1.115 |
| Porosity (%) | 85.2 |
| Total intrusion volume (mL/g) | 5.25 |

**Figure S8.** Pore size distribution and textural data by Hg intrusion porosimetry for Ni0.07/Ce0.9La0.1.

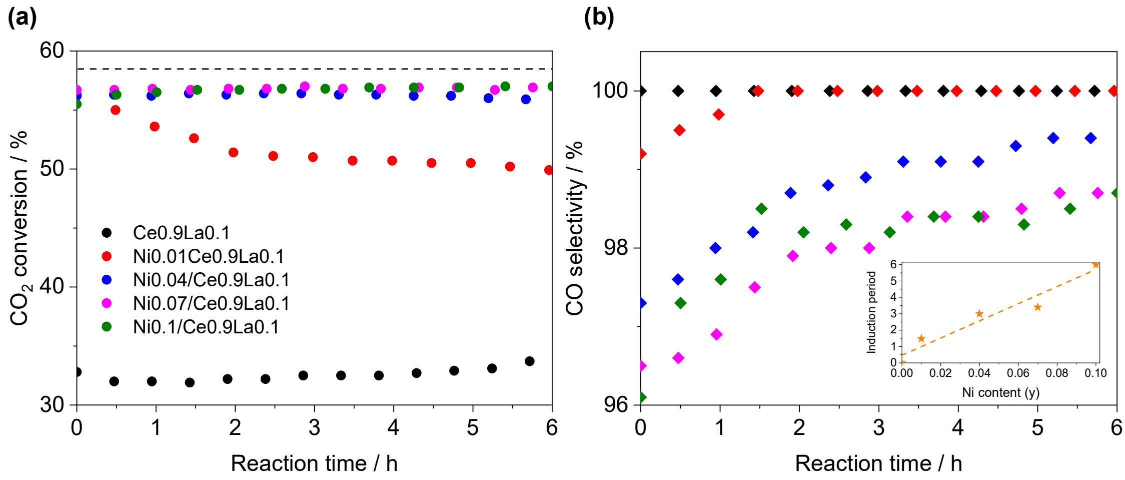


**Figure S9.** Catalytic performance of $Ni_y/(Ce_{0.9}La_{0.1}O_{1.95})_{1-y}$ for the rWGS reaction (700ºC, $H_2/CO_2$=2 (molar), $3\cdot10^5$ $mL_N\cdot h^{-1}$ gram of catalyst$^{-1}$). (a) $CO_2$ conversion vs reaction time. (b) CO selectivity vs reaction time, inset: Induction period vs Ni molar proportion, "y".

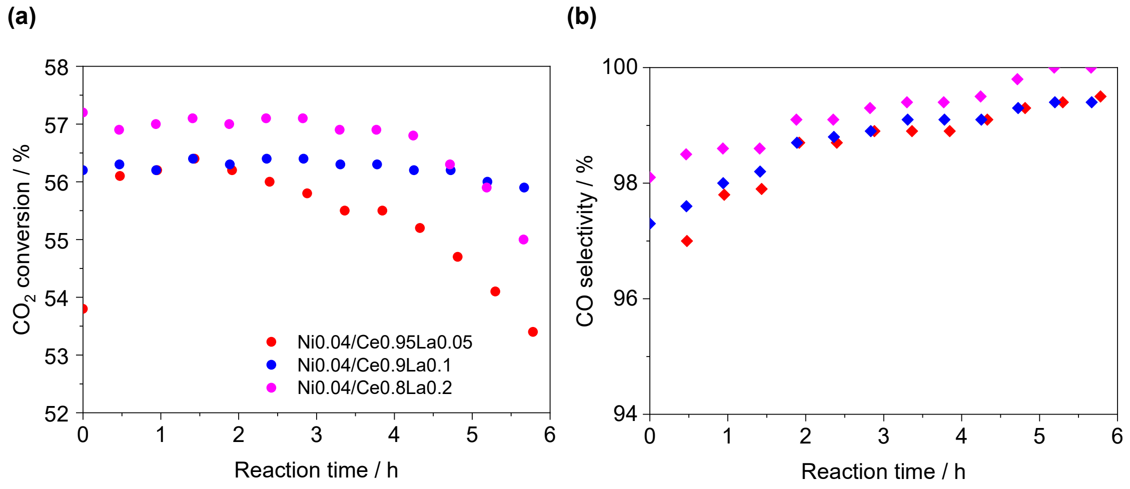


**Figure S10**. Catalytic performance of $Ni_{0.04}(Ce_{1-x}La_xO_{2-x/2})_{0.96}$ series for the rWGS reaction (700ºC, $H_2/CO_2=2$ (molar), $3\cdot10^5$ $mLN\cdot h^{-1}$gram of catalyst$^{-1}$). (a) $CO_2$ conversion vs reaction time. (b) CO selectivity vs reaction time.

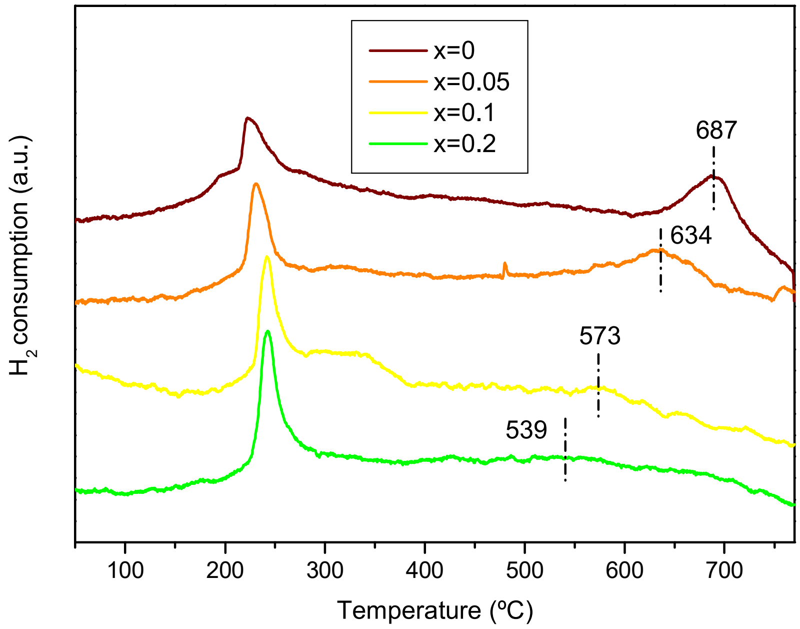


**Figure S11.** Temperature reduction profiles of fresh prepared $Ni_{0.07}/(Ce_{1-x}La_xO_{2-x/2})_{0.93}$.

The redox properties of the catalysts have been studied by $H_2$-Temperature Programmed Reduction on the fresh prepared $Ni_{0.07}/(Ce_{1-x}La_xO_{2-x/2})_{0.93}$ catalysts for a constant Ni loading and a varying proportion of La proportion. The more defined peaks centered around 250°C are assigned to the reduction of the NiO overlayer that passivates metallic Ni particles, whereas the $H_2$ consumption between 300–400°C is related to the reduction of a $NiO_x$ phase that have a greater interaction with the support, i.e. at the metal-ceria interface. The low consumption in the range 400–550 °C is explained by the reduction of some $Ni^{2+}$ species within the ceria support. As reported in literature, the last reduction peak (550–800ºC) is ascribed to the surface and bulk reduction of ceria (at lower and higher temperature, respectively.[9,10]

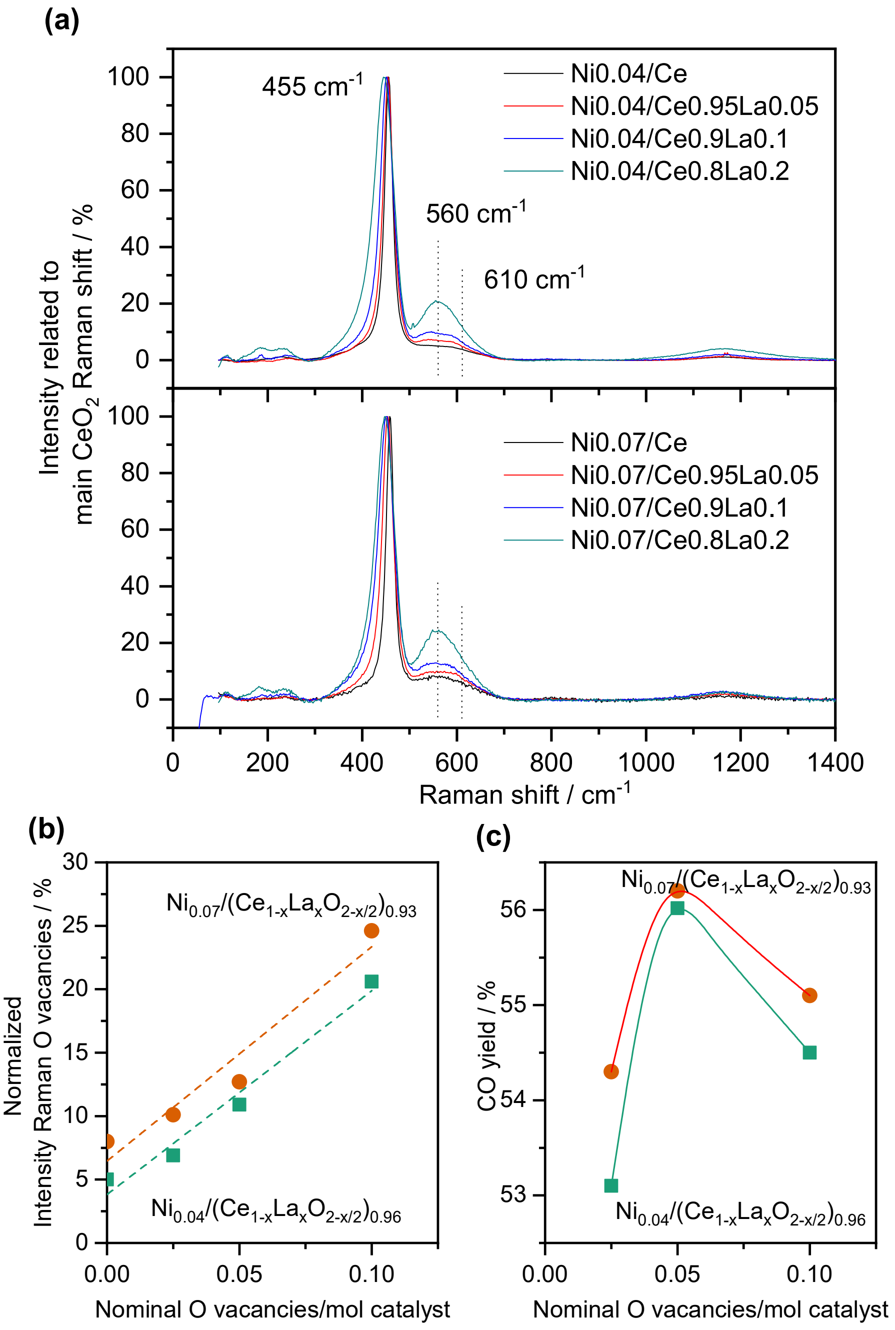


**Figure S12.** a) Raman spectra for $Ni_y/(Ce_{1-x}La_xO_{2-x/2})1\text{-}y$, (x=0, 0.05, 0.1, 0.2; y = 0.04, 0.07) series, b) Normalized Raman shift intensity for 560 $cm^{-1}$ (extrinsic oxygen vacancies) in relation to main Ce-O vibration at 455 $cm^{-1}$ versus nominal oxygen vacancies, c) CO yield (after 6 hours of reaction) versus nominal oxygen vacancies (per catalyst mol) for $Ni_{0.04}/(Ce_{1-x}La_xO_{2-x/2})_{0.96}$ and $Ni_{0.07}/(Ce_{1-x}La_xO_{2-x/2})_{0.93}$.

Figure S12a depicts a Raman spectra for $Ni_{0.04}/(Ce_{1-x}La_xO_{2-x/2})_{0.96}$ and $Ni_{0.07}/(Ce_{1-x}La_xO_{2-x/2})_{0.93}$ (x= 0, 0.05, 0.1, 0.2) catalysts after reaction, and Figure S12b shows the correlation between the nominal concentration of oxygen vacancies for each catalyst versus the corresponding normalized intensity of the Raman shift related to the extrinsic oxygen vacancies, that logically increases with La proportion. Moreover. Figure S12c illustrates the CO yield (after 6 hours of reaction) versus the nominal concentration of oxygen vacancies (per catalyst mol), which shows a volcano plot, i.e., the catalytic activity for the rWGS reaction passes through a maximum as function of the oxygen vacancy concentration.

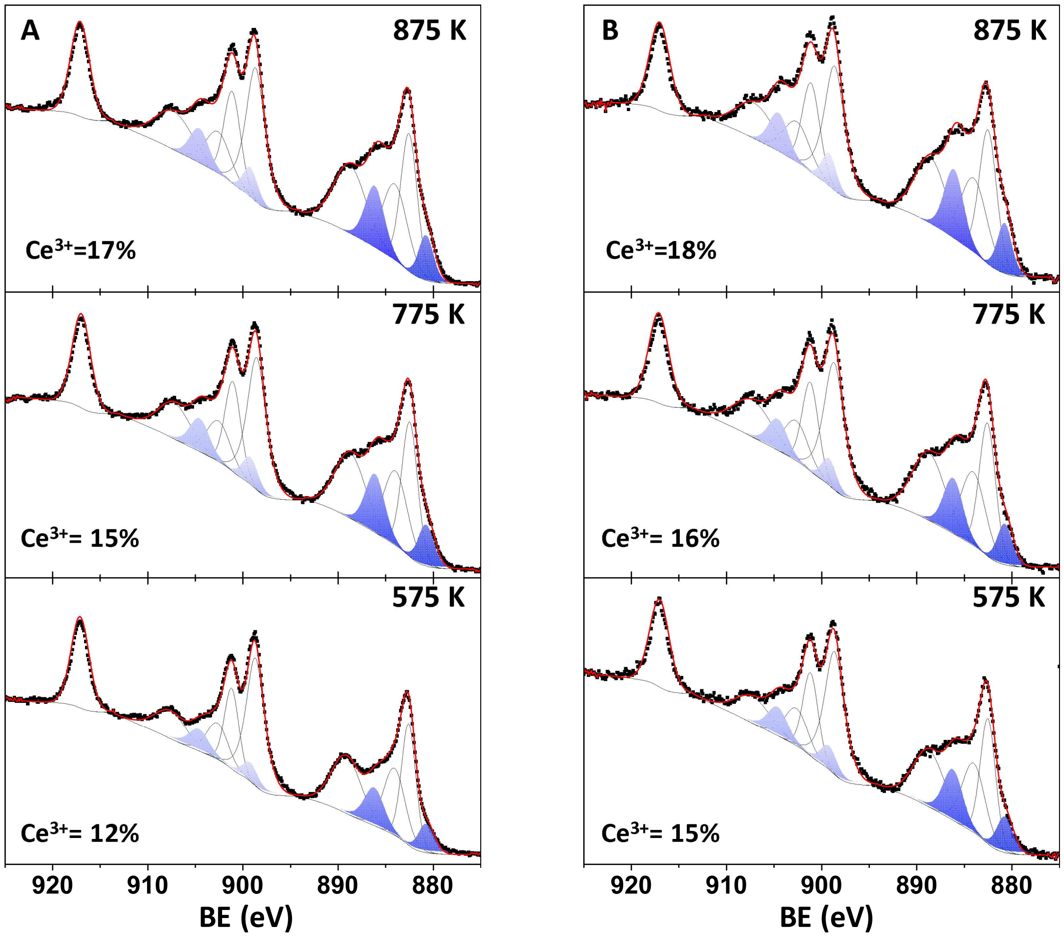


**Figure S13.** NAP-XPS of Ce3d region for A) Ni0.07/Ce and B) Ni0.07/Ce0.8La0.2 under $H_2$:$CO_2$= 2 at different temperatures ($Ce^{3+}$ 3d5/2 and $Ce^{3+}$ 3d3/2 levels in violet and light violet color, respectively).

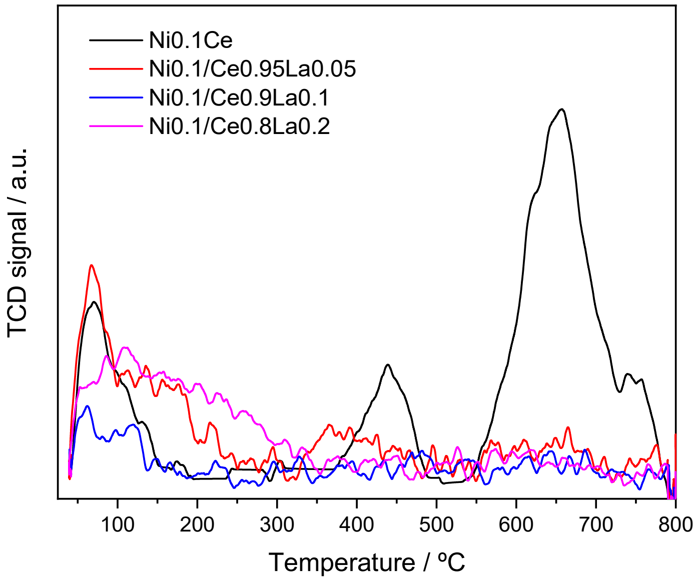


**Figure S14.** $CO_2$-Temperature programmed desorption ($CO_2$-TPD) profiles of Ni/$CeO_2$ and Ni/La-doped $CeO_2$ based catalysts

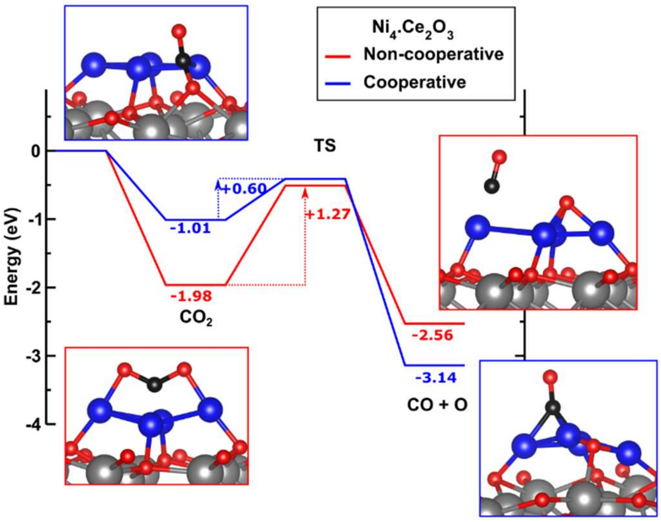


**Figure S15.** Cooperative and non-cooperative reaction pathways for $CO_2$ dissociation on $Ni_4.Ce_2O_3$.

The structures shown to the left and right of the reaction pathways correspond to the side views of the optimized molecularly initial and final states used in the search of the transition state structure. All energies are relative to $CO_2$ in the gas phase. Atom color code: nickel atoms are depicted in blue, oxygen in red, carbon in black, hydrogen in yellow and $Ce^{3+}$ in gray.

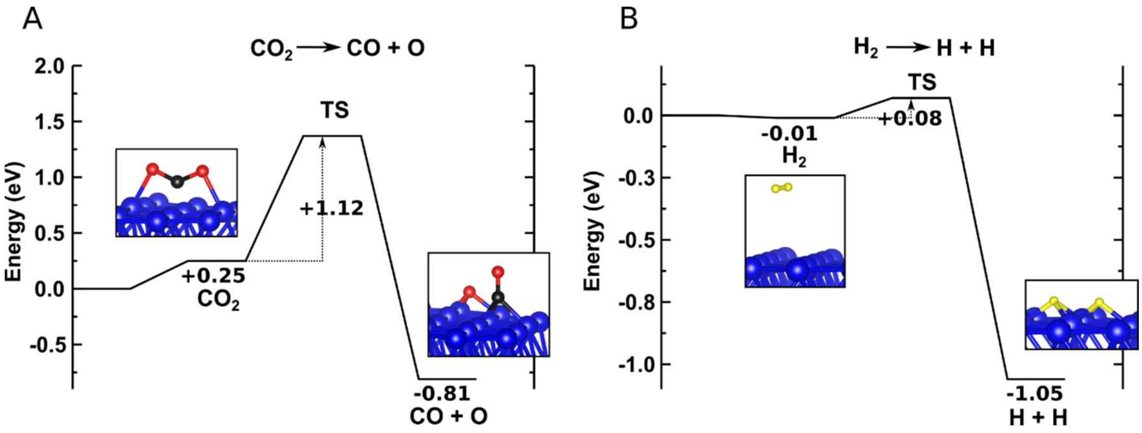


**Figure S16.** Reaction pathways for $CO_2$ and $H_2$ dissociation on Ni(111).

Reaction pathways for (A) $CO_2$ and (B) $H_2$ dissociation on Ni(111). The structures shown to the left and right of the reaction pathways correspond to the side views of the optimized molecularly initial and final states used in the search of the transition state structure. All energies are relative to (A) $CO_2$ and (B) $H_2$ in the gas phase. Atom color code: nickel atoms are depicted in blue, oxygen in red, hydrogen in yellow.

## References


1. Kim, S. S.; Lee, H. H. and Hong, S. C., A Study on the Effect of Support's Reducibility on the Reverse Water-Gas Shift Reaction over Pt Catalysts, *Appl. Catal. A*, **2012**, *423–424*, 100–107.
2. Zhang, Y.; Liang, L.; Chen, Z.; Wen, J.; Zhong, W.; Zou, S.; Fu, M.; Chen L. and Ye, D., Highly Efficient Cu/$CeO_2$-Hollow Nanospheres Catalyst for the Reverse Water-Gas Shift Reaction: Investigation on the Role of Oxygen Vacancies through in situ UV-Raman and DRIFTS, *Appl. Surf. Sci.*, **2020**, *516*, 146035.
3. L. Wang, H. Liu, Y. Liu, Y. Chen and S. Yang, Influence of Preparation Method on Performance of Ni-$CeO_2$ Catalysts for Reverse Water-Gas shift Reaction, *J. Rare Earths*, **2013**, *31*, 559–564.
4. Zonetti, P. C., Letichevsky, S., Gaspar, A. B.; Sousa- Aguiar, E. F. and Appel, L. G., The $Ni_xCe_{0.75}Zr_{0.25-x}O_2$ Solid Solution and the RWGS, *Appl. Catal. A*, **2014**, *475*, 48–54.
5. Vogt, C. *et al.* Understanding Carbon Dioxide Activation and Carbon-Carbon Coupling over Nickel. *Nat Commun.,* **2019**, 10, 5330, doi: 10.1038/s41467-019-12858-3.
6. Lu, X. *et al.* Initial Reduction of $CO_2$ on Perfect and O-Defective $CeO_2$ (111) Surfaces: towards CO or COOH? *RSC Advances,* **2015**, 5, 97528-97535.
7. Padama, A. A. B., Kasai, H.; Kawai, H. The Mechanism of $H_2$ Dissociation and Adsorption on Mn-modified Ni(111) Surface: A Density Functional Theory-based Investigation. *Surf. Sci.*, **2012**, 606, 62-68.

8. Fernandez-Torre, D.; Carrasco, J.; Ganduglia-Pirovano, M. V.; Perez, R. Hydrogen Activation, Diffusion, and Clustering on $CeO_2$(111): a DFT+U Study. *J Chem. Phys.,* **2014**, *141*, 014703, doi:10.1063/1.4885546.
9. Pino, L., Vita, A., Cipiti, F., Lagana, M. and Recupero, V. Hydrogen Production by Methane tri-Reforming Process over Ni-ceria Catalysts: Effect of La-doping. *Appl. Catal. B: Environ.*, **2011**, *104*, 64-73.
10. Alvarez-Galvan, M. C. *et al.* Performance of La,Ce-Modified Alumina-Supported Pt and Ni Catalysts for the Oxidative Reforming of Diesel Hydrocarbons. *Int. J. Hydrog. Energy*, **2008**, *33*, 652-663.
